# Persimmon leaf extract in dyslipidemia: a systematic review and meta-analysis

**DOI:** 10.3389/fphar.2025.1572678

**Published:** 2025-09-15

**Authors:** Chenxuan Dong, Tianying Chang, Xiaoli Wang, Lei Zhou, Lisha Wang, Xiaodan Wang, Yu Sun, Xing Liao, Yingzi Cui, Jiajuan Guo

**Affiliations:** ^1^ College of Traditional Chinese Medicine, Changchun University of Chinese Medicine, Changchun, China; ^2^ EBM Office, The Affiliated Hospital of Changchun University of Chinese Medicine, Changchun, China; ^3^ Department of Cardiology, The Affiliated Hospital of Changchun University of Chinese Medicine, Changchun, China; ^4^ Department of Pathology, The Affiliated Hospital of Changchun University of Chinese Medicine, Changchun, China; ^5^ Institute of Clinical Basic Medicine of Chinese Medicine, China Academy of Chinese Medical Sciences, Beijing, China; ^6^ President’s Office, The Affiliated Hospital of Changchun University of Chinese Medicine, Changchun, China

**Keywords:** persimmon leaf extract, dyslipidemia, systematic review, meta-analysis, *Diospyros kaki Thunb*. [Ebenaceae, *Kaki folium*]

## Abstract

**Aim:**

This study aims to evaluate the effects of the combined application of *Diospyros kaki* Thunb. [Ebenaceae; *Kaki folium*] (Persimmon leaf) extract on lipid profiles in adults and to explore its potential role in preventing and treating lipid disorders and associated diseases, including common comorbidities such as cardiovascular and cerebrovascular diseases, as well as medication-induced dyslipidemia.

**Methods:**

From the inception of the database to 1 November 2024, we retrieved randomized controlled trials (RCTs) from eight English and Chinese databases.

**Results:**

A total of 704 articles were retrieved, from which 16 studies were selected for inclusion in the systematic review and meta-analysis. These studies encompassed 1,572 patients, with 790 assigned to the treatment group and 782 to the control group. Among the dyslipidemia patients included in the study, the most common comorbidities were coronary artery disease, hypertension, ischemic cerebrovascular disease, and dyslipidemia induced by olanzapine, among others. Compared with the control group, the use of combined Persimmon leaf extract (PLE) significantly reduced total cholesterol (TC), triglycerides (TG), and low-density lipoprotein cholesterol (LDL-C) levels, while demonstrating a certain degree of improvement in high-density lipoprotein cholesterol (HDL-C) levels.

**Conclusion:**

PLE has been shown to be effective in improving blood lipid profiles in patients, suggesting its potential for widespread clinical application. However, the significant heterogeneity observed across existing studies, coupled with the frequent occurrence of methodological flaws, emphasizes the need for well-designed clinical trials with large sample sizes and extended follow-up periods.

**Systematic Review Registration:**

https://www.crd.york.ac.uk/PROSPERO/display_record.php?RecordID=562090, identifier CRD42024562090.

## 1 Introduction

Diospyros represents the largest genus in the Ebenaceae family. The genus Diospyros is widely distributed across East Asia, including China, Korea, and Japan. It is also cultivated in countries such as India, Azerbaijan, Spain, Turkey, Brazil, Mexico (Ramírez-Briones et al., 2019), and the United States (Hossain and Shahidi, 2023). The edible and medicinal properties of its fruit and leaves have contributed to Diospyros gaining increasing recognition and widespread utilization in traditional medicine and the food industry across various regions. *Diospyros kaki* is regarded as the most promising species (Butt et al., 2015; Rauf et al., 2017; Fareed et al., 2022). The fruit of this species can be consumed fresh or processed, while the leaves have an even broader range of applications. Persimmon leaf tea has demonstrated anti-aging properties (Sakanaka et al., 2005). Additionally, due to their antibacterial and health-promoting properties, persimmon leaves are incorporated as food additives in cookies, sushi, rice cakes, and other foods (Kim et al., 2005). Persimmon leaves have a long history of use in China, as well as in other countries such as Spain, India, Japan, and South Korea. The medicinal use of persimmon leaves in China was first recorded in 1436 AD in Lan Mao’s *Southern Yunnan Materia Medica* (Su, 1436), and traditional Chinese medicine (TCM) has since accumulated extensive experience.

Persimmon leaves contain a variety of flavonoids, including quercetin, kaempferol, myricetin, vitexin (Hong et al., 2023), proanthocyanidins, and flavonol glycosides, alongside other phenolic metabolites, terpenoids, and polysaccharides such as cellulose, hemicellulose, and lignin, among other bioactive metabolites (Ramírez-Briones et al., 2019). Consequently, they exhibit a broad spectrum of pharmacological activities, encompassing anti-atherosclerotic effects, cardiovascular protection, hypoglycemic and hypolipidemic actions, anti-inflammatory, antibacterial, antioxidant, anticancer, anti-allergic, antihypertensive, neuroprotective, and hemostatic properties. Currently, persimmon leaves are chiefly utilized in clinical treatments for conditions such as atherosclerosis, ischemic angina pectoris, stroke, internal bleeding, hypertension, and other conditions, as well as for frostbite, burns, and constipation, among other symptoms (Xie et al., 2015; Hossain and Shahidi, 2023).

Naoxinqing Tablet (NXQT) is a commercial Chinese polyherbal preparation (CCPP) containing *Diospyros kaki* Thunb. [Ebenaceae; *Kaki folium*] (Persimmon leaf) extract, with each tablet weighing 0.41 g and containing 100 mg of Persimmon leaf extract (PLE). Due to its extensive clinical applications, NXQT has been included in the Pharmacopoeia of the People’s Republic of China (National Drug Code Z20053259). Its primary metabolites include flavonoids, triterpenoids, and phenolic acids, with flavonoids constituting 25.7% of the total content and being abundant in various flavonoid metabolites, such as hypericin, isoquercitrin, astragalin, quercetin, and kaempferol (Huang et al., 2016; Kazzem et al., 2019; Liu, 2023). Studies have demonstrated that quercetin and kaempferol, the primary bioactive flavonoids, are the key therapeutic metabolites of NXQT (Luo et al., 2004). It is widely used in clinical practice for the treatment of various cardiovascular and cerebrovascular diseases, including ischemic cerebrovascular diseases, epilepsy, coronary heart disease with angina pectoris, hypertension, chronic heart failure, lower extremity arteriosclerosis obliterans, antipsychotic drug-induced dyslipidemia, and primary hyperlipidemia. An expert consensus has also been published on the use of NXQT to treat ischemic cerebrovascular diseases (Wang, 2023). Since then, the extract derived from persimmon leaves has been further developed into various dosage forms, including notably Naoxinqing Capsule (NXQC, National Drug Code Z20050673) for clinical use (Zhang, 2021; Jiang et al., 2024).

Animal studies have demonstrated that applying PLE can lower the levels of various plasma lipid indices (Zhang et al., 2005; Lee et al., 2006), likely through the modulation of key genes and proteins involved in lipid metabolism, such as fatty acid synthase (FAS), stearoyl-CoA desaturase 1 (SCD1), and others (Chen et al., 2011a; Jung et al., 2012). A substantial body of evidence indicates that dyslipidemia is a pivotal pathogenic factor in numerous diseases, including atherosclerotic cardiovascular disease (ASCVD), stroke, non-alcoholic fatty liver disease, and metabolic disorders (Corey and Chalasani, 2014; Aradine et al., 2020; Mach et al., 2020; Arvanitis and Lowenstein, 2023). Research has demonstrated that various lipids and lipoproteins significantly influence the incidence and progression of cardiovascular diseases (CVD) (Bhargava et al., 2022). The findings indicate that elevated levels of low-density lipoprotein cholesterol (LDL-C) are a primary cause of ischemic heart disease and stroke in both developed and developing countries. In 2019, approximately 4.4 million deaths and 98.62 million disability-adjusted life years (DALYs) were attributed to high plasma LDL-C levels (Pirillo et al., 2021). In 2021, 3.81 million cardiovascular deaths were attributed to elevated LDL-C levels (Vaduganathan et al., 2022). Additionally, epidemiological studies have demonstrated an increased incidence of cardiovascular events in patients with high triglyceride (TG) levels (Klempfner et al., 2016; Nichols et al., 2019). Recent research has shown that Lp(a) independently increases the risk of ASCVD and that effective reduction of LDL-C levels does not counteract the Lp(a)-mediated risk (Bhatia et al., 2024). Furthermore, numerous national guidelines for preventing and treating CVD, as well as predictive models for cardiovascular risk assessment, explicitly underscore the importance of managing various lipid indicators (Arnett et al., 2019; Cardiovascular Disease Branch of the Chinese Medical Association et al., 2020; Visseren et al., 2021; Wong et al., 2022). Abnormalities in blood lipid levels are frequently associated with a wide range of diseases.

Dyslipidemia management primarily involves lifestyle modifications and pharmacological therapies. Lifestyle modifications encompass adopting a balanced diet, increasing physical activity moderately, managing weight, quitting smoking, and moderating alcohol consumption. When lifestyle interventions fail to achieve target lipid levels, lipid-lowering medications are recommended, with statins being the cornerstone of cholesterol-lowering therapy. Statins are widely recommended for dyslipidemia treatment and are extensively used in both primary and secondary prevention of CVD (Vaduganathan et al., 2022). Despite their potent lipid-lowering effects, statins are associated with several adverse events. Numerous studies have reported statin-associated adverse effects, primarily including liver injury and a spectrum of muscle-related complications, such as myalgia, myositis, myopathy, and rhabdomyolysis (Stroes et al., 2015). Additional adverse effects include an increased risk of new-onset type 2 diabetes mellitus (T2DM) (Ruscica et al., 2014), statin intolerance (Cheeley et al., 2022), a heightened risk of adverse reactions when combined with other medications (Lamprecht et al., 2022), as well as symptoms such as insomnia, depression, and gastrointestinal discomfort. Studies have demonstrated that statin utilization is also lower among individuals with elevated risk factors, which is presumed to be associated with the absence of health insurance that covers the expenses of long-term, high-intensity statin therapy (Pirillo et al., 2021). Furthermore, other therapeutic options exhibit specific adverse effects, such as Omega-3 fatty acids potentially inducing atrial fibrillation and flutter adverse events and gemfibrozil within the fibrate class, possibly increasing the risk of gallstones and cholecystitis (Newman, 2022; Wang et al., 2023b). Therefore, the current medications available for the treatment and prevention of dyslipidemia remain inadequate. TCM treatments have gradually been incorporated into the comprehensive management of dyslipidemia, providing a beneficial supplement to conventional biomedicine. This integration has been recommended and recognized in national lipid management guidelines, further highlighting its significant role and importance in treating dyslipidemia.

In recent years, determining the metabolites in persimmon leaves has advanced, leading to a more precise elucidation of its pharmacological effects. The efficacy and underlying mechanisms of the combined application of PLE in treating dyslipidemia have been substantiated not only in numerous animal experiments but also in several clinical trials. However, a comprehensive synthesis of clinical evidence regarding the effectiveness of PLE in treating dyslipidemia is still lacking. Existing human studies have notable limitations, such as small sample sizes, inconsistent clinical outcomes, and insufficient statistical power to detect moderate effects. Therefore, it is essential to conduct a systematic review and meta-analysis of the available clinical data to evaluate the therapeutic efficacy of PLE in improving blood lipid levels. Given the harmful impact of dyslipidemia on health, the proven effectiveness of PLE in treating this condition, and the lack of a systematic review that quantitatively summarizes its clinical effects, this meta-analysis aims to comprehensively evaluate the current evidence on the use of PLE for treating dyslipidemia. Furthermore, by analyzing the heterogeneity among studies, we aim to provide insights that may guide future investigations.

## 2 Methods

### 2.1 Protocol and registration

The research proposal has been registered with the International Prospective Register of Systematic Reviews (PROSPERO) (Registration ID: CRD42024562090), and this study adhered to the 2020 guidelines of the Preferred Reporting Items for Systematic Reviews and Meta-Analyses (PRISMA).

### 2.2 Search strategy

RCTs investigating the treatment of dyslipidemia with PLE were retrieved from Chinese databases (CNKI, VIP, Wanfang Data, SinoMed) and English databases (PubMed, EMBASE, Cochrane Library, Web of Science). Publications in both Chinese and English were included, spanning from the inception of these databases to November 2024, with no restrictions on publication date or language. The search terms used included: “randomized controlled trial,” “Persimmon leaf flavonoids,” “persimmon leaf extract,” “persimmon leaf,” and “Naoxinqing”; the specific search strategy can be found in Supplementary Table 1. To ensure that no relevant literature was omitted, a manual search was performed on the references of the included studies, related review articles, and clinical trial registries. Authors were not contacted for additional information, and unpublished studies were not considered.

### 2.3 Study selection

Using the PICOS framework as a template, we identified the general characteristics of the included trials, which encompass participants, interventions, comparisons, outcomes, and study designs, as illustrated in Table 1. To ensure the methodological rigor and quality of the study, only research reports meeting the following inclusion criteria were considered: (1) The randomized controlled trial (RCT) aims to explore the effects of PLE on lipid profiles in patients with various conditions, including but not limited to hypertension, coronary artery disease, cerebrovascular diseases, and lipid abnormalities induced by olanzapine administration; (2) The intervention should consist of PLE, derived from the species *Diospyros kaki* (Family: Ebenaceae), and administered either alone or in combination with other biomedicines for the treatment of dyslipidemia. The extract should be prepared using established methods, such as those used in the commercial formulations NXQT and NXQC, which involve standardized extraction and processing techniques to preserve the active metabolites; (3) A control group was included; (4) The treatment duration was at least 2 weeks; (5) The studies reported levels of at least one of the following lipid indicators: total cholesterol (TC), LDL-C, high-density lipoprotein cholesterol (HDL-C), and TG; (6) They provided the mean ± standard deviation of baseline and final values, along with the 95% confidence interval (CI) for the outcomes.

**TABLE 1:** Inclusion criteria for PICO framework.

Criteria	Description
Population	Adults (aged >18 years)
Intervention	The intervention involved PLE or NXQT (or NXQC), administered for ≥2 weeks
Comparison	The control group underwent treatment adhering to the standardized biomedicine therapy as recommended by the pertinent guideline
Outcome	Mean values and standard deviations (SD) of TC, TG, HDL-C and LDL-C, along with adverse events, were reported for both intervention and control groups
Study design	RCTs

In addition, studies were excluded if they met any of the following criteria: (1) *In vitro* or animal experiments; (2) Non-randomized controlled trials, cohort studies, case reports, literature reviews, meeting abstracts, or studies with inappropriate control or intervention groups; (3) Studies lacking sufficient data or with unclear presentations in the articles. In cases of duplicated studies, we only included the most recent or the most comprehensive ones.

Two independent investigators performed reference screening, reviewed the titles and abstracts of retrieved publications, and conducted full-text evaluations of potentially eligible studies to determine their eligibility based on the predefined inclusion and exclusion criteria. To ensure the accuracy of the study selection process, calibration exercises were conducted, and consensus was reached with the assistance of an additional researcher in case of any disagreements. These methods and criteria enhanced the reliability and validity of the meta-analysis.

### 2.4 Data extraction

Standardized data collection forms were employed to extract the following details from eligible studies: reference source, first author, publication year, study location, study design, sample size, patient population characteristics (e.g., gender and health status), treatment details (including the dosage form, dose, frequency of administration, and treatment cycle of PLE), treatment protocols of the control group, baseline an outcome indicators with their mean ± standard deviation (SD), and adverse events. Two investigators independently performed data extraction and cross-checked the literature search, data extraction, and bias assessment processes. Any discrepancies were resolved through discussion or consultation with a third investigator.

### 2.5 Quality assessment

The quality of each included study was assessed using the Cochrane Collaboration’s tool for risk of bias (RoB) (Higgins et al., 2011). This tool is specifically designed to assess seven critical aspects of each trial comprehensively, including the generation of a random sequence; allocation concealment; blinding of participants and personnel; blinding of outcome assessors; handling of incomplete outcome data; selective reporting; and other potential biases. Risks associated with each aspect are categorized as low risk, risk of uncertain significance, or high risk. The final decision was made after reaching unanimous consent among all investigators.

### 2.6 Data synthesis and statistical analysis

Statistical data were analyzed using Review Manager version 5.4 software. Control results for lipid levels were assessed through TC, TG, HDL-C, and LDL-C measurements, and the changes in mean and standard deviation (SD) values between baseline and endpoint in both the treatment and control groups were tabulated. Post-intervention values and the number of participants for each study were entered into Review Manager 5.4 to calculate the standardized mean difference (SMD). The fixed-effects model was applied when the heterogeneity index (I^2^) is less than 50% (Higgins et al., 2003). Conversely, a random-effects model is utilized if the I^2^ value is greater than or equal to 50% (DerSimonian and Laird, 1986). When at least ten studies reported outcomes, publication bias was assessed via a funnel plot. Sensitivity analysis was conducted by sequentially removing individual studies to evaluate the robustness of the meta-analysis. Subgroup analyses were performed based on dosage form and dose to assess the impact of these variables on the efficacy of PLE in treating dyslipidemia.

### 2.7 Quality of evidence and evaluation of this SR

Quality evaluations were conducted utilizing the Grading of Recommendations Assessment, Development and Evaluation (GRADE) guidelines to assess the certainty of evidence for each outcome, encompassing five critical areas of assessment: (1) Evaluating study limitations by the Risk of Bias in Systematic Reviews 2.0 (RoB2.0); (2) Assessing consistency using the I^2^ value and examining the agreement between the 95% confidence interval and prediction interval; (3) Evaluating the directness of evidence in terms of whether the interventions and populations included in the studies are pertinent to the research question; (4) Assessing precision based on the optimal information sample size; (5) Evaluating publication bias utilizing funnel plots and taking into account the number of included studies. Furthermore, the methodological quality and risk of bias in the meta-analysis, independently conducted by two investigators without any conflict of interest, were assessed using the enhanced Systematic Review Quality Assessment Scale (AMSTAR-2) (Shea et al., 2017).

## 3 Results

### 3.1 Study selection

**FIGURE 1:**
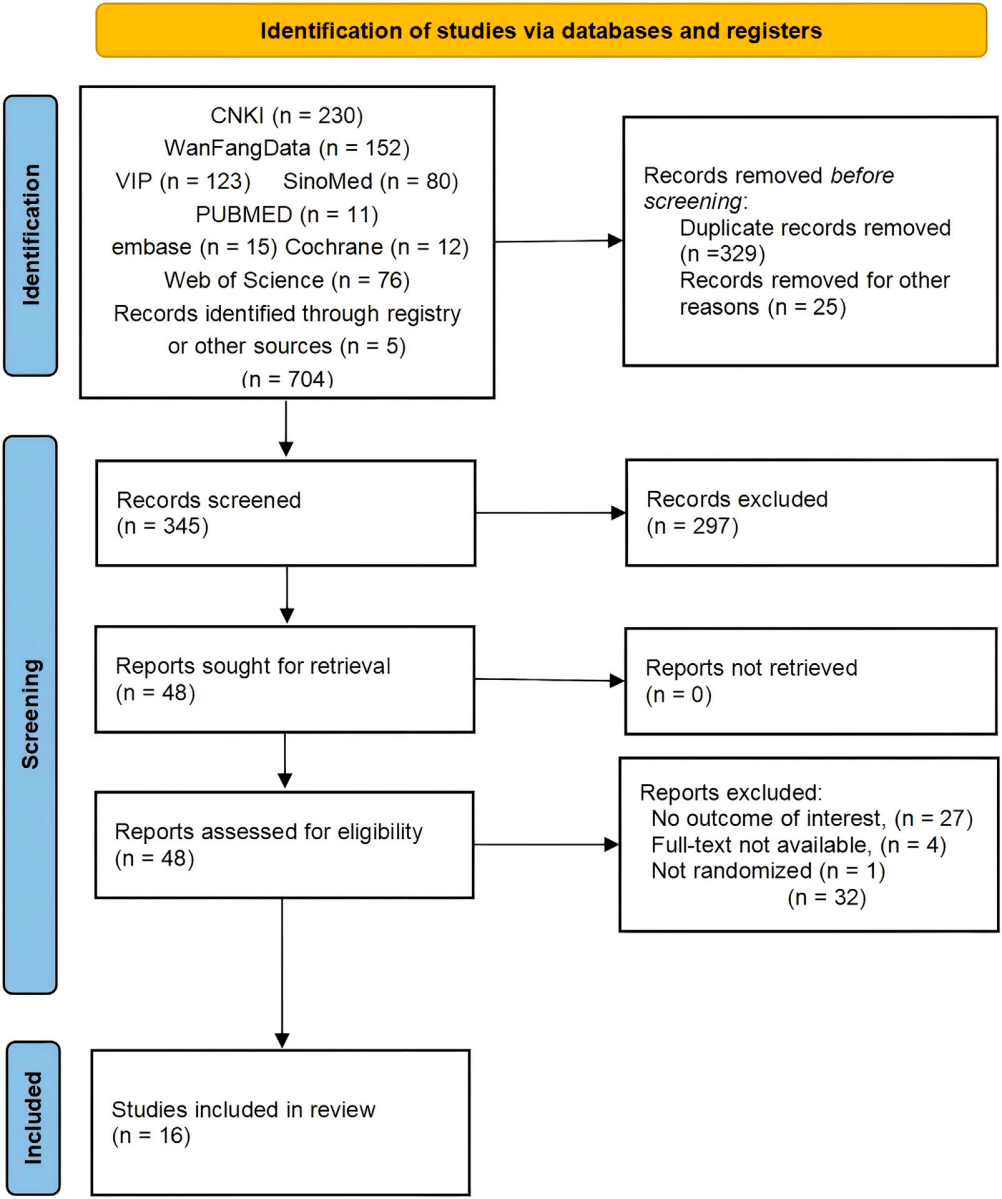
PRISMA flow-chart of study selection.

The process of selecting the included studies and the specific reasons for excluding articles is illustrated in the PRISMA flowchart (Figure 1). From the eight databases mentioned above, 699 articles were retrieved. Additionally, supplementary searches of clinical trial registries (ClinicalTrials.gov, WHO-ICTRP) and reference lists identified five additional eligible studies, resulting in 704 unique records for screening. After removing duplicates, 345 articles remained for further evaluation. After screening the titles and abstracts, we excluded 297 papers that did not meet the inclusion criteria. The remaining 48 articles were assessed through full-text review. Among these, 32 studies were excluded due to various reasons. Ultimately, 16 studies were included in the meta-analysis.

### 3.2 Study characteristics

The detailed baseline characteristics of the 16 studies are presented in Table 2. Sixteen studies, conducted between 2004 and 2024 (Wu, 2004; Wu et al., 2008; Tang et al., 2012; Huang, 2013; Qiao et al., 2013; Xiong et al., 2013; Yang et al., 2014; Zhou et al., 2015; Wang, 2016; Zhao, 2016; Hong et al., 2017; Wei et al., 2018; Liu, 2019; Lv et al., 2020; Zhang, 2021; Jiang et al., 2024), were published in China. The patient enrollment in these studies ranged from 41 to 200, totaling 1,572 participants. Of these participants, 790 were allocated to the treatment group and 782 to the control group. Within populations affected by dyslipidemia-related conditions, research was conducted in the following subpopulations: four studies focused on patients with coronary heart disease (Wu, 2004; Qiao et al., 2013; Xiong et al., 2013; Jiang et al., 2024), three in those with ischemic cerebrovascular disease (Tang et al., 2012; Zhou et al., 2015; Lv et al., 2020), two in hypertensive patients (Huang, 2013; Yang et al., 2014), two in individuals with hyperlipidemia (Wu et al., 2008; Wei et al., 2018), two in patients with schizophrenia (Hong et al., 2017; Liu, 2019), one in those with chronic cerebral insufficiency (Zhao, 2016), one in cases of cerebral arteriosclerosis (Zhang, 2021), and one in patients suffering from lower extremity arteriosclerosis (Wang, 2016). The control group was treated with standard pharmacological therapies appropriate for their respective conditions, such as valsartan tablets, atorvastatin calcium tablets, aspirin tablets, metoprolol sustained-release tablets, isosorbide dinitrate tablets, and fluvastatin capsules. Among the studies, thirteen utilized NXQT manufactured by Guangzhou Baiyunshan Hutchison Whampoa Chinese Medicine Co., LTD., two used NXQC produced by Shenyang Dongxin Pharmaceutical Co., LTD., and one did not report the manufacturer. The duration of intervention ranged from 2 to 48 weeks. The commercial preparation summary table and the inclusion/exclusion criteria for the studies included in this research are presented in Supplementary Tables 2 and 3.

**TABLE 2:** Characteristics of the included trials.

Study	Year	Clinical trial design	Population	Intervention treatment group (per dose)	Control group	Sample size (I/C)	Study period	Outcome measure
Hong	2017	RCT	Dyslipidemia due to olanzapine^a^	Olanzapine tablet 5 mg + NXQ 1.23 g	Olanzapine tablet 5 mg	43/43	24 weeks	①②③④
Huang	2013	RCT	Hypertension^b^	Valsartan capsule 80 mg + NXQ 1.64 g	Valsartan capsule 80 mg	60/60	4 weeks	①②④
Jiang	2024	RCT	Stable angina pectoris^c^	Metoprolol succinate sustained release tablets 23.75 mg/day + NXQ capsule 0.9 g	Metoprolol succinate sustained release tablets 23.75 mg/d	55/55	8 weeks	①②③④
Liu	2019	RCT	Dyslipidemia due to olanzapine^a^	Olanzapine tablet 5–20 mg + NXQ 0.82–1.64 g	Olanzapine tablet 5–20 mg	25/25	12 weeks	①②
Lv	2020	RCT	Acute cerebral infarction^d^	Atorvastatin 10 mg +NXQ 0.82 g	Atorvastatin 10 mg	53/53	24 weeks	④
Qiao	2013	RCT	Coronary heart disease^m^	Aspirin 100 mg +Atorvastatin 20 mg + Metoprolol succinate sustained release tablets 25 mg + isosorbide dinitrate10 mg + NXQ 1.64 g	Aspirin 100 mg +Atorvastatin 20 mg +metoprolol extended-release tablet 25 mg +isosorbide dinitrate 10 mg	85/83	48 weeks	①②③④
Tang	2012	RCT	Diabetic cerebral infarction^m^	Basic treatment + NXQ 1.64 g	Basic treatment	35/33	6 weeks	①②③④
Wang	2016	RCT	Lower extremity atherosclerotic occlusive disease^e^	Fluvastatin capsules 20/40 mg + NXQ 0.82–1.64 g	Fluvastatin capsules 20/40 mg	47/47	12 weeks	①
Wei	2018	RCT	Hyperlipidemia^f^	Basic treatment + NXQ 1.23 g	Basic treatment	60/60	24 weeks	①②③④
Wu	2004	RCT	Coronary heart disease^g^	Basic treatment + NXQ 0.82	Basic treatment	37/30	4 weeks	①②
Wu	2008	RCT	Hyperlipidemia^h^	Simvastatin tablets 20 mg + NXQ 1.23 g	Simvastatin tablets 40 mg	19/22	8 weeks	①②③④
Xiong	2013	RCT	Unstable angina pectoris^m^	Basic treatment + NXQ (Unstated dose)	Basic treatment	60/60	2 weeks	①②③④
Yang	2014	RCT	Hypertension^i^	Valsartan 80 mg/day + NXQ 1.23 g	Valsartan 80 mg/d	30/30	24 weeks	①②③④
Zhang	2021	RCT	Cerebral arteriosclerosis^j^	Basic treatment + NXQ capsule 1.2 g	Basic treatment	50/50	4 weeks	①②③④
Zhao	2016	RCT	Chronic cerebral circulatory insufficiency^k^	Cinrizine tablets 25 mg + NXQ 0.82 g	Cinrizine tablets 25 mg	31/31	4 weeks	①②
Zhou	2015	RCT	Acute ischemic cerebral disease^l^	Basic treatment +NXQ 1.23 g	Basic treatment	100/100	24 weeks	①②③④

①TG; ②TC; ③HDL-C; ④LDL-C.

a. The International Classification of Diseases, 10th Edition (ICD-10), Classification of Mental and Behavioral Disorders; b. Diagnosis and classification of hypertension; c. Guidelines for the diagnosis and treatment of stable coronary heart disease; d. 2010 Chinese Guidelines for the Diagnosis and Treatment of Acute Ischemic Stroke; e. The Latest Comprehensive Book on Traditional Chinese Medicine for Peripheral Vascular Diseases; f. Guidelines for the Prevention and Treatment of Dyslipidemia in Chinese Adults; g. WHO’s diagnostic criteria for coronary heart disease and the “Diagnostic Criteria for Coronary Heart Disease and Angina Pectoris” revised at the National Conference on Coronary Heart Disease and Angina Pectoris; h. Guidelines for Clinical Research on New Traditional Chinese Medicines; i. World Health Organization (WHO)/International Society of Hypertension (ISH) diagnostic criteria; j. Exploration of Cerebral Arteriosclerosis and Its Early Diagnosis; k. CCCI, Diagnostic Criteria Established at the 2000 Japan Stroke Conference; l. Standards of the fourth National Cerebrovascular Disease Academic Conference (1995); m. Not described.

### 3.3 Preparation of persimmon leaf extract

All the studies included in our research exclusively utilized the NXQT or NXQC, with PLE as the sole active constituent. Both formulations are approved by Chinese drug regulatory authorities and have been commercially available for several years.

#### 3.3.1 Naoxinqing tablet

The extraction process of the NXQT is that the persimmon leaves are extracted, separated, and purified to obtain the acetate ethyl ester extract of persimmon leaves. Then, suitable excipients including starch, sucrose powder, magnesium stearate and microcrystalline cellulose, etc., are added, mixed evenly, granulated, dried, pressed into tablets, and coated with a film.

#### 3.3.2 Naoxinqing capsule

NXQC is prepared by decocting dried persimmon leaves twice in water—first with 12 volumes of water for 2 h, and then with 10 volumes for 1 h. The combined filtrate is filtered and concentrated to a relative density of 1.12–1.15 at 60 °C. Ethanol is then added to achieve an alcohol content of 85%. The mixture is left to stand overnight, and the supernatant is filtered for further processing. The resulting precipitate is washed twice with four volumes of 65% ethanol, and the eluate is collected and allowed to stand. The ethanol is recovered, and the resulting extract is dissolved in 2 times the volume of water, filtered, and the filtrate is extracted with (3×, 2×, 2×, and 1×) volumes of ethyl acetate. The combined organic phase is recovered and evaporated at 60 °C to yield a dry extract. This is blended with starch (100 g starch per 50 g extract) to form a moist mass, which is then granulated, dried at 60 °C, and encapsulated to produce the final product.

### 3.4 Quality assessment

The studies were evaluated using the GRADE approach for treatment assessment, as presented in Table 3, and heterogeneity was noted in all of the studies. The quality of evidence for TC was rated as very low, whereas the evidence quality for TG, LDL-C, and HDL-C was rated as low. The pooled results demonstrated that the mean difference (MD) for TC decreased by 0.91 (95% CI: 1.26 to 0.55), TG decreased by 0.43 (95% CI: 0.62 to 0.24), LDL-C decreased by 0.47 (95% CI: approximately 0.60 to 0.34), and HDL-C increased by 0.24 (95% CI: 0.13–0.35).

**TABLE 3:** GRADE summary of finding.

Quality assessment							No of patients		Effect		Quality	Importance
No of studies	Design	Risk of bias	Inconsistency	Indirectness	Imprecision	Other considerations	Serum lipid parameter	Control	Relative (95% CI)	Absolute		
TC (Better indicated by lower values)												
14	Randomised trials	Serious^1^	Serious^2^	No serious indirectness	Serious^3^	None	670	667	-	MD 0.91 lower (1.26–0.55 lower)	⊕ΟΟΟ VERY LOW	CRITICAL
TG (Better indicated by lower values)												
15	Randomised trials	Serious^1^	Serious^2^	No serious indirectness	No serious imprecision	None	717	714	-	MD 0.43 lower (0.62–0.24 lower)	⊕⊕ΟΟ LOW	CRITICAL
LDL-C (Better indicated by lower values)												
12	Randomised trials	Serious^1^	Serious^2^	No serious indirectness	No serious imprecision	None	630	634	-	MD 0.47 lower (0.6–0.34 lower)	⊕⊕ΟΟ LOW	CRITICAL
HDL-C (Better indicated by lower values)												
10	Randomised trials	Serious^1^	Serious^2^	No serious indirectness	No serious imprecision	None	517	521	-	MD 0.24 higher (0.13–0.35 higher)	⊕⊕ΟΟ LOW	CRITICAL

Question: serum lipid parameter for dyslipidemia.

^1^ Most studies exhibited inadequate descriptions of randomization and were consequently deemed to possess a high risk of bias, due to their failure to elucidate whether concealed grouping and blinding techniques were employed.

^2^ A notably high I-squared value indicates the presence of considerable heterogeneity among the studies.

^3^ The sample size was comparatively modest, and the confidence interval extended to an effect size exceeding 0.5 on one tail.

**FIGURE 2:**
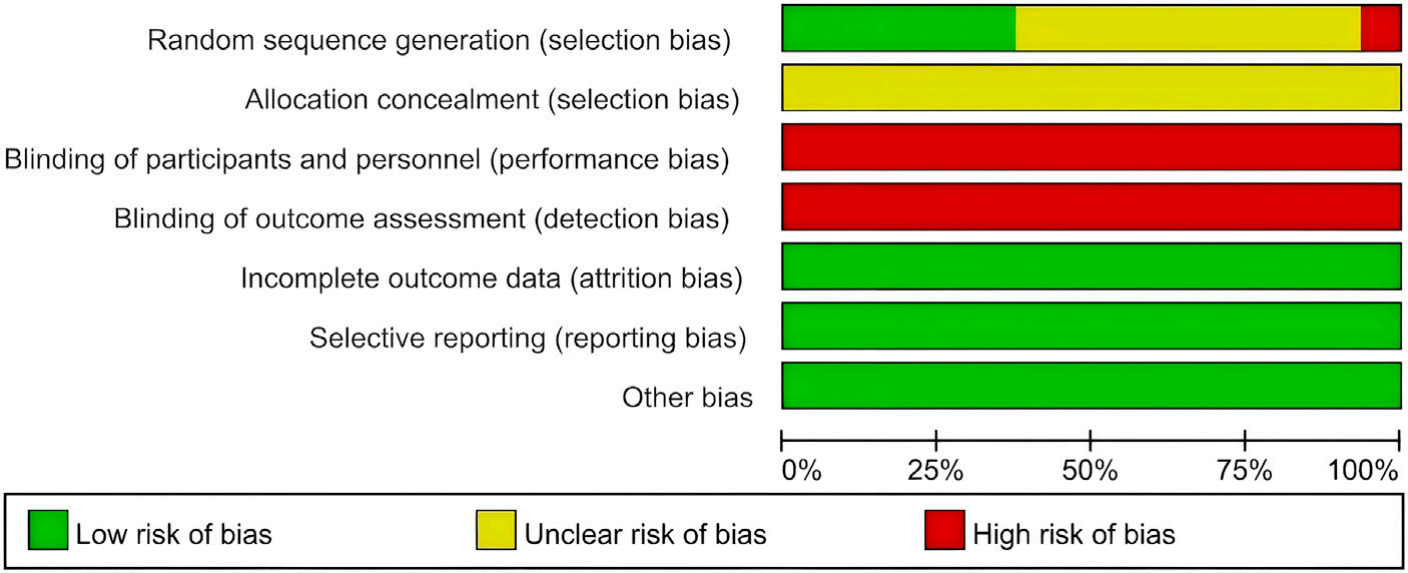
Risk of bias graph.

**FIGURE 3:**
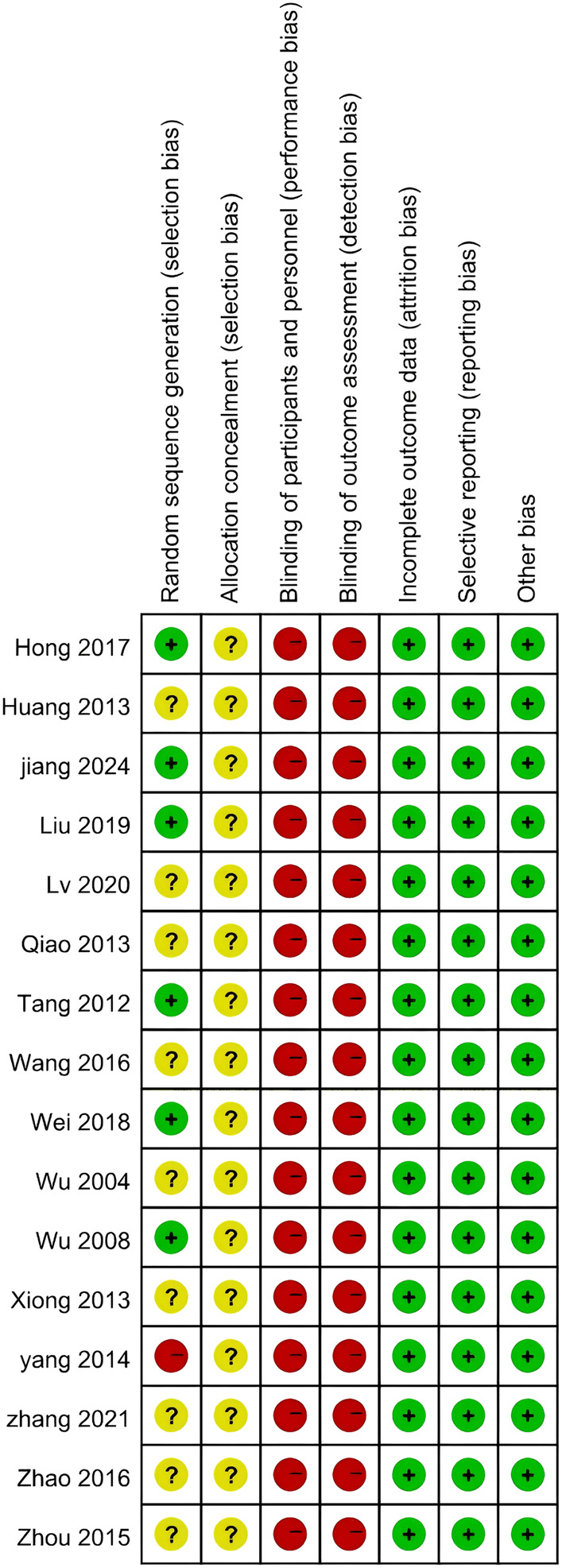
Risk of bias summary.

Six RCTs demonstrated a low risk of bias in “random sequence generation” (Wu et al., 2008; Tang et al., 2012; Hong et al., 2017; Wei et al., 2018; Liu, 2019; Jiang et al., 2024), while one study exhibited a high risk (Yang et al., 2014), and the remaining studies presented an unclear risk of bias. Notably, none of the studies mentioned the methods of “allocation concealment”, resulting in an unclear appraisal for these domains. Since all the studies employed a non-blind design, the “Blinding of participants and personnel” and “Blinding of outcome assessment” items were consistently rated as high risk across all studies. All studies showed a low risk of bias regarding “incomplete outcome data”, “selective reporting” and “Other bias”. The systematic assessment of bias is illustrated in Figures 2, 3. Moreover, this study was assessed using the AMSTAR 2 and PRISMA 2020 checklists, with the results presented in Supplementary Table 4. Funnel plots corresponding to the results of the four clinical outcomes are shown in Supplementary Figure 1.

### 3.5 Meta-analysis

#### 3.5.1 TC

**FIGURE 4:**
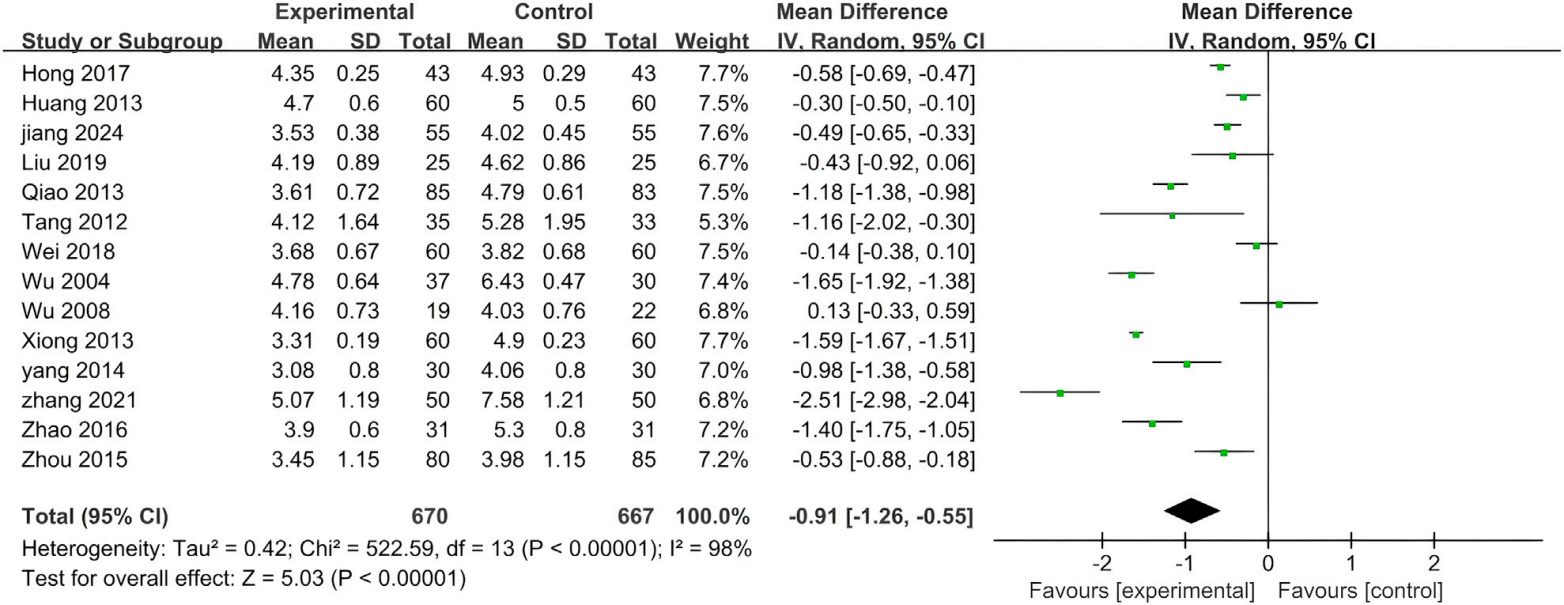
A forest plot comparing PLE combination therapy with standardized treatment for TC. The experimental group comprised patients receiving PLE in addition to standardized treatment, whereas the control group consisted solely of patients undergoing standardized treatment.

Among the 14 studies, TC was chosen as the primary outcome measure. Given the significant heterogeneity (p < 0.00001, I^2^ = 98%), a random effects model was employed to analyze the gathered data. As depicted in Figure 4, when compared to standardized treatment alone, the results of this meta-analysis indicate that combined application of PLE effectively reduces TC levels in patients (MD = −0.91; 95% CI: −1.26 to −0.55; Z = 5.03, p < 0.00001).

**FIGURE 5:**
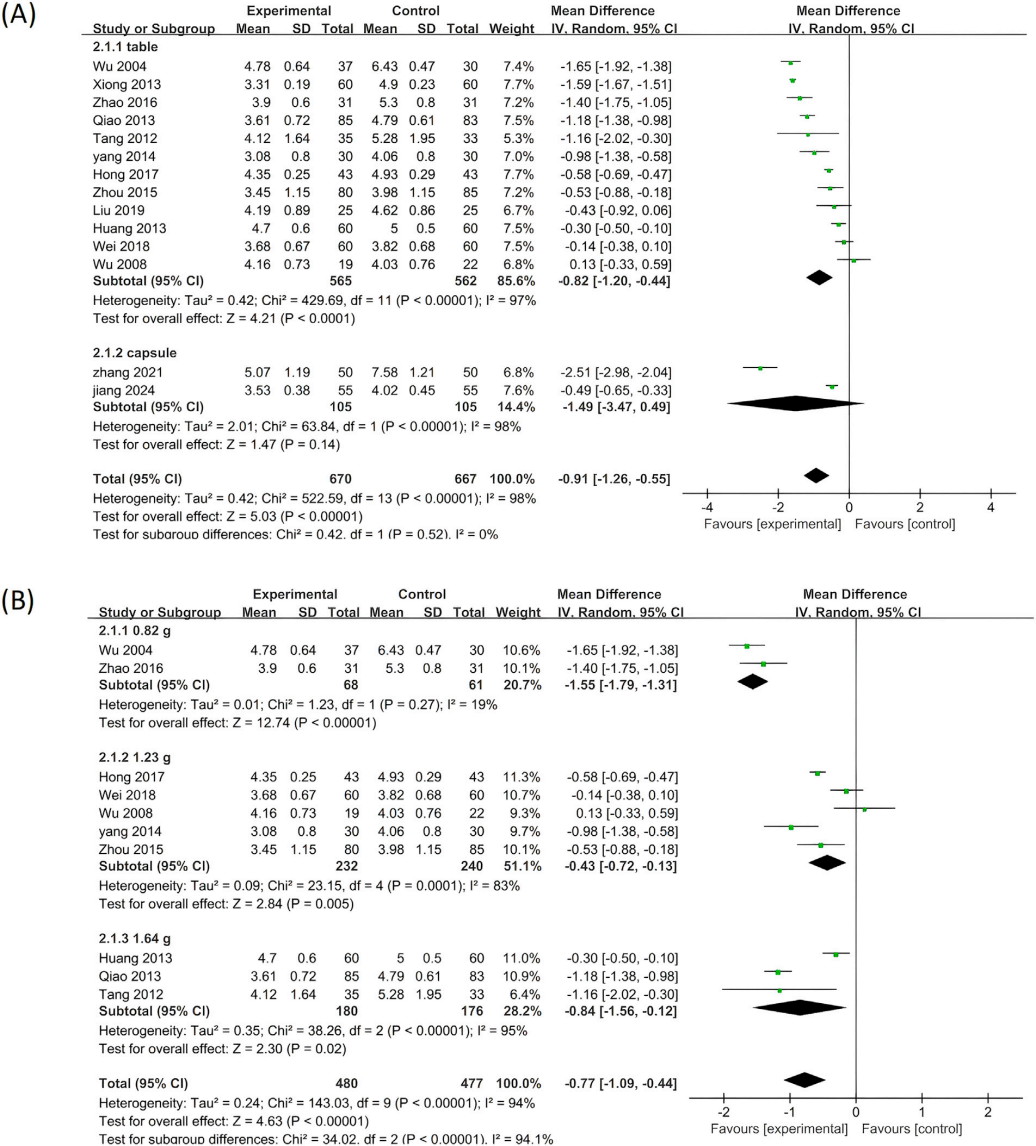
Subgroup analysis of the TC. **(A)** Stratified by the form of a drug. **(B)** Stratified by drug dose.

Further subgroup analyses were conducted based on variations in drug dosage forms and doses to explore the influence of these variables on the therapeutic effect of PLE (Figure 5). These analyses revealed that, although a minority of subgroups did not exhibit a significant positive effect, they did not detract from the overall outcomes. When stratified by dosage forms, the combination therapy with NXQC showed no significant impact on reducing TC levels (MD = −1.49; 95% CI: −3.47 to 0.49; Z = 1.47, p = 0.14). Conversely, when stratified by drug doses, the application of PLE in combination therapy demonstrated a significant positive effect in reducing TC levels, with the most significant therapeutic effect observed in the 0.82 g dose group (MD = −1.55; 95% CI: −1.79 to −1.37; Z = 12.47, p < 0.00001).

#### 3.5.2 TG

**FIGURE 6:**
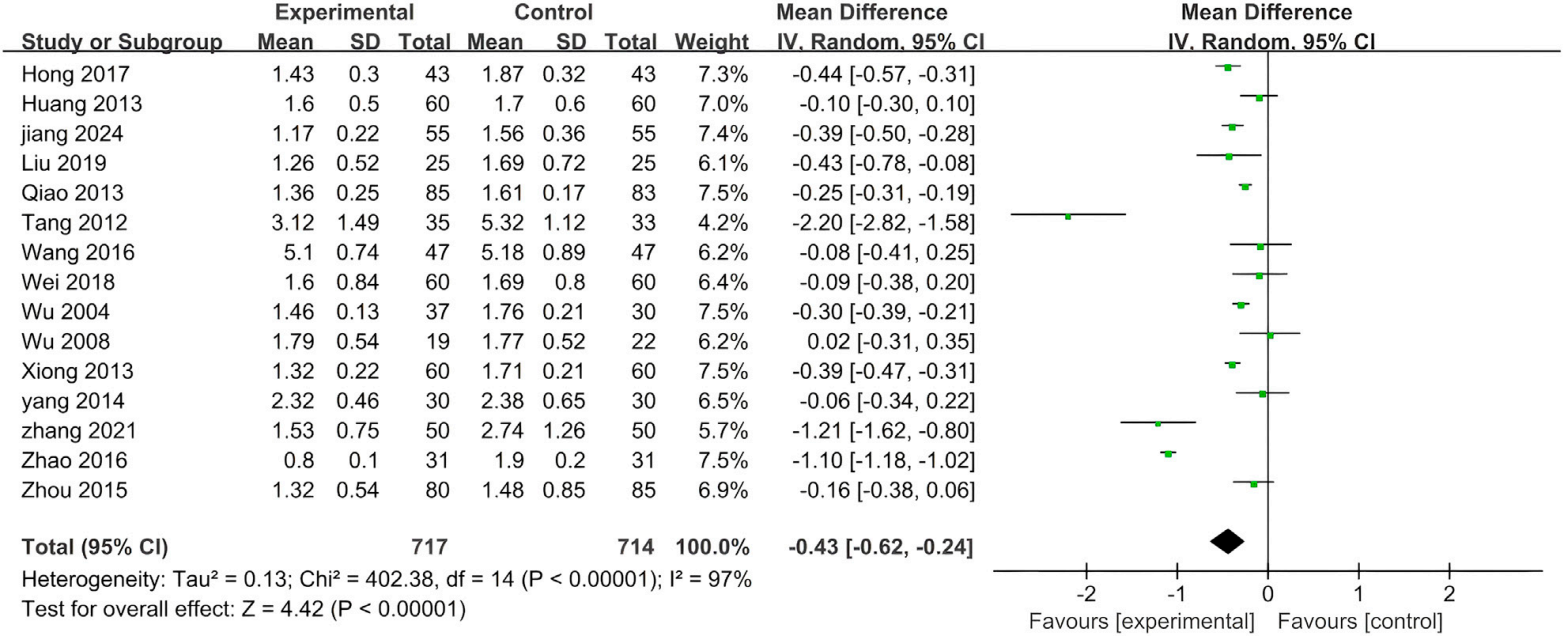
A forest plot comparing PLE combination therapy with standardized treatment for TG. The experimental group comprised patients receiving PLE in addition to standardized treatment, whereas the control group consisted solely of patients undergoing standardized treatment.

TG was utilized as a detection index in 15 studies. As depicted in Figure 6, a random effects model was adopted for the analysis due to the significant heterogeneity observed among the 15 studies (p < 0.00001, I^2^ = 97%). The findings indicated that the combined application of PLE, in conjunction with the standardized treatment, could more effectively diminish the level of TG (MD = −0.43; 95% CI: −0.62 to −0.24; Z = 4.42, p < 0.00001).

**FIGURE 7:**
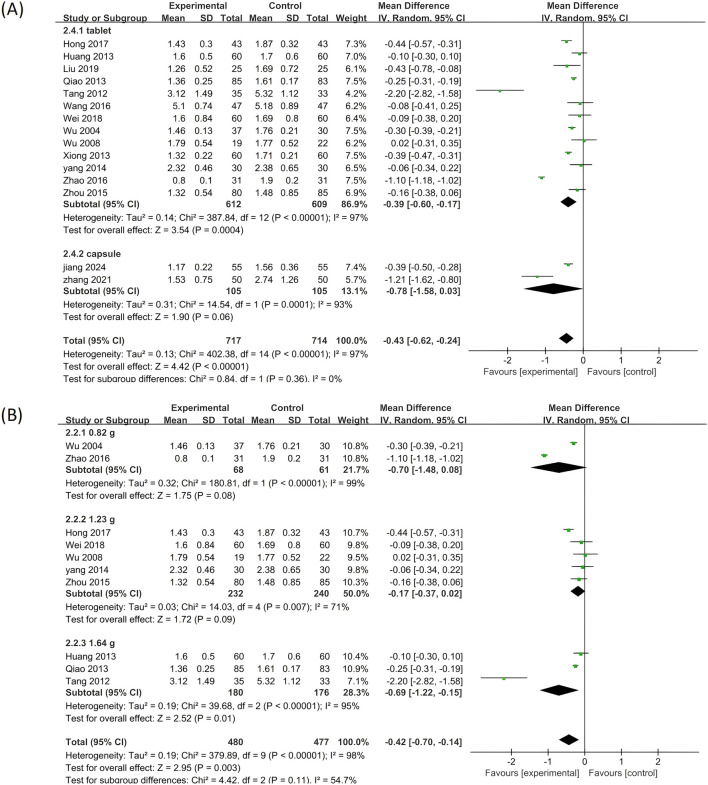
Subgroup analysis of the TG. **(A)** Stratified by the form of a drug. **(B)** Stratified by drug dose.

The results of the subgroup analysis indicated that the combination therapy with NXQT mirrored the overall conclusion (Figure 7), whereas the combination of capsule formulations with standardized treatment failed to reduce TG levels (MD = −0.78; 95% CI: −1.58 to 0.03; Z = 1.90, p = 0.06). Specifically, at doses of 0.82 g (MD = −0.70; 95% CI: −1.48 to 0.08; Z = 1.75, p = 0.08) and 1.23 g (MD = −0.17; 95% CI: −0.37 to 0.02; Z = 1.72, p = 0.09), no statistically significant therapeutic effect on lowering TG levels was observed. Conversely, a statistically significant therapeutic effect was noted at a dose of 1.64 g per administration (MD = −0.69; 95% CI: −1.22 to −0.15; Z = 2.52, p = 0.01).

#### 3.5.3 LDL-C

**FIGURE 8:**
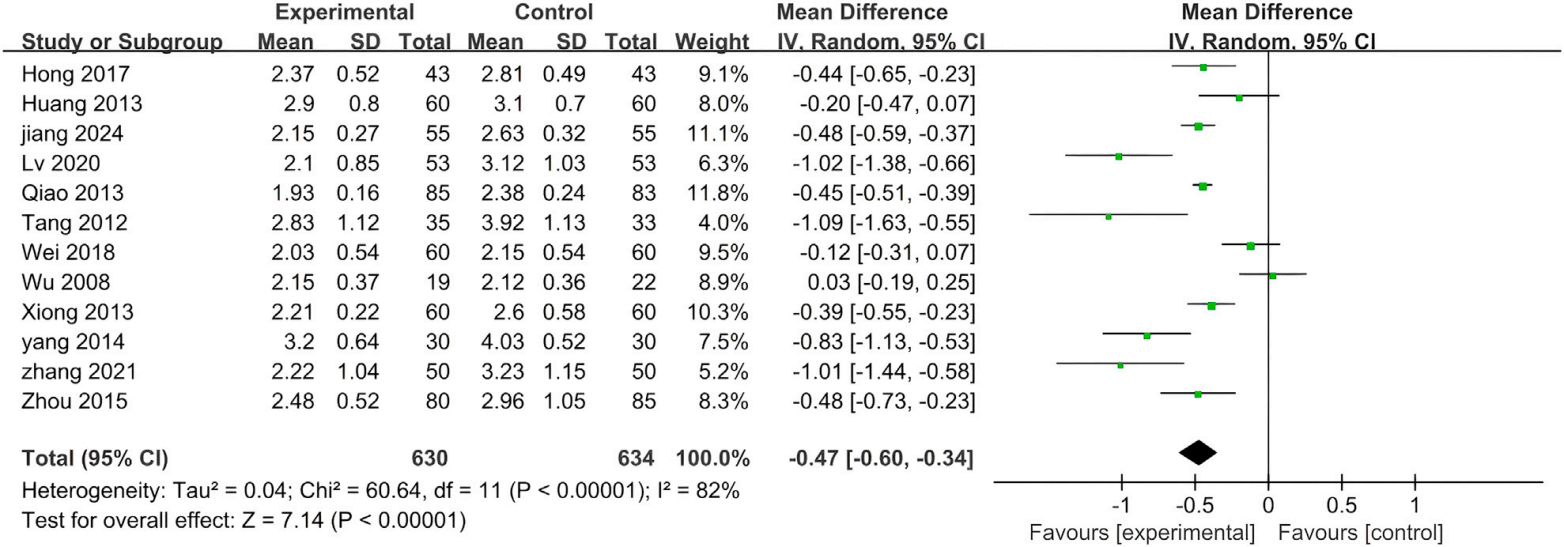
A forest plot comparing PLE combination therapy with standardized treatment for LDL-C. The experimental group comprised patients receiving PLE in addition to standardized treatment, whereas the control group consisted solely of patients undergoing standardized treatment.

Twelve of the included clinical studies reported LDL-C as an outcome measure. As depicted in Figure 8, significant heterogeneity was observed among the twelve studies (p < 0.00001, I^2^ = 82%), prompting the adoption of the random effects model in this analysis. Compared to standardized treatment, adding PLE as combination therapy significantly reduced the LDL-C levels (MD = −0.47; 95% CI: −0.60 to −0.34; Z = 7.14, p < 0.00001).

**FIGURE 9:**
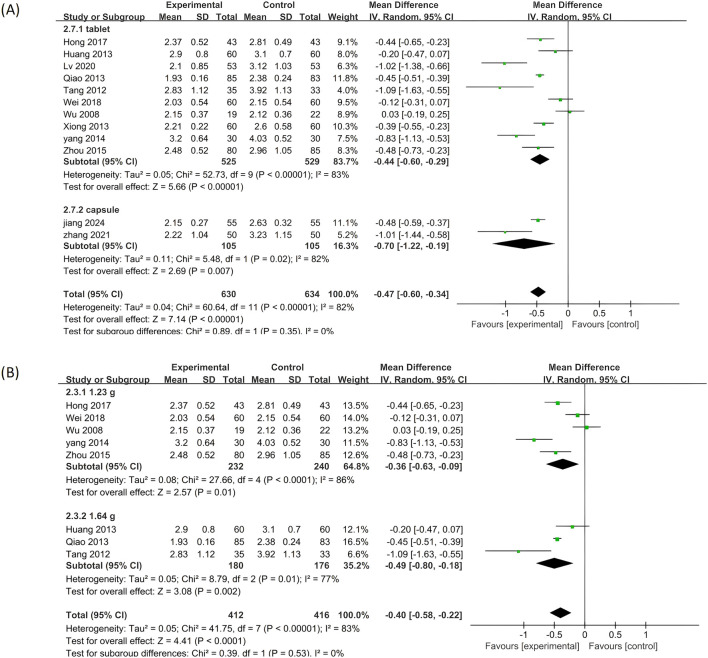
Subgroup analysis of the LDL-C. **(A)** Stratified by the form of a drug. **(B)** Stratified by drug dose.

Further subgroup analysis was conducted, and the findings across all subgroups were in line with the overall results (Figure 9), indicating that these variables did not exert a significant impact on the reduction of LDL-C levels achieved by PLE.

#### 3.5.4 HDL-C

**FIGURE 10:**
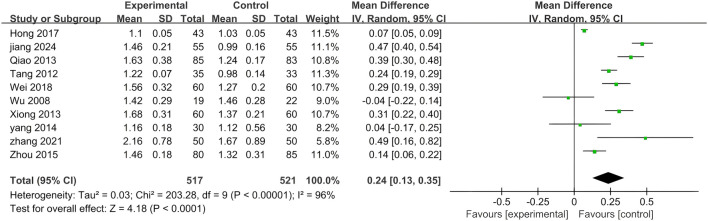
A forest plot comparing PLE combination therapy with standardized treatment for HDL-C. The experimental group comprised patients receiving PLE in addition to standardized treatment whereas the control group consisted solely of patients undergoing standardized treatment.

A total of ten included studies utilized HDL-C as an outcome measure. The results of this analysis are presented in Figure 10. Notably, significant differences were observed among the seven studies (p < 0.00001, I^2^ = 96%), necessitating the application of the random effects model for analysis in this study. The findings indicated that adding PLE to treatment was more effective in managing HDL-C levels than treatment with biomedicine alone (MD = 0.24; 95% CI: 0.13 to 0.35; Z = 4.18, p < 0.0001).

**FIGURE 11:**
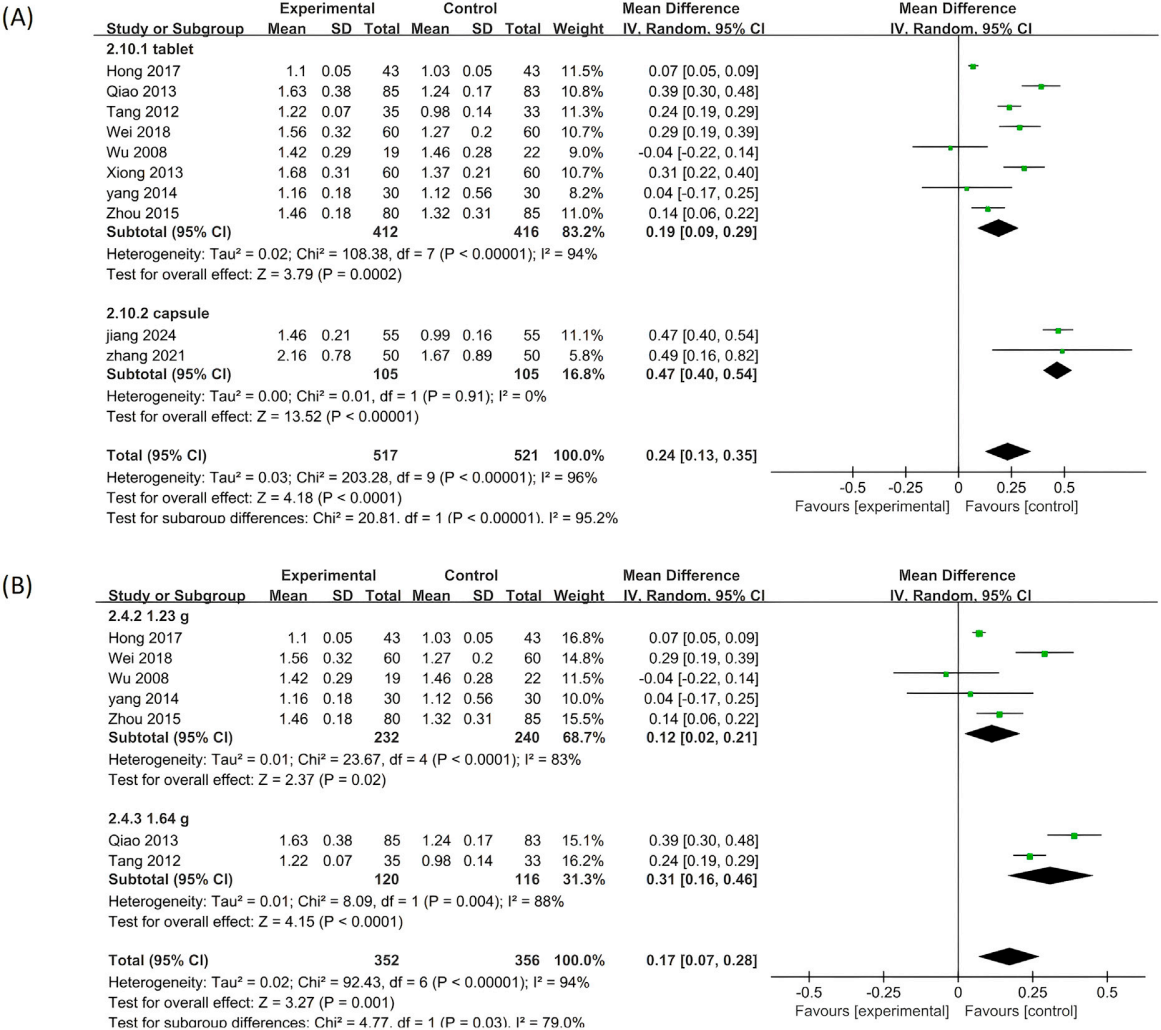
Subgroup analysis of the HDL-C. **(A)** Stratified by the form of a drug. **(B)** Stratified by drug dose.

A subgroup analysis was conducted to explore the potential influence of various parameters on the effectiveness of PLE in enhancing HDL-C levels (Figure 11). The results from the subgroup analysis of different dosage forms within the combination therapy were consonant with the overall findings, demonstrating statistically significant positive effects.

### 3.6 Adverse reaction

Adverse effects were reported in 11 studies, while no adverse events were documented in the remaining studies. Among the 11 studies that reported adverse effects (Wu, 2004; Wu et al., 2008; Tang et al., 2012; Huang, 2013; Xiong et al., 2013; Yang et al., 2014; Wang, 2016; Zhao, 2016; Hong et al., 2017; Zhang, 2021; Jiang et al., 2024), involving a total of 928 patients, the incidence of adverse events was marginally lower in the treatment group compared to the control group. Specifically, four studies reported no adverse events in either the treatment or control group (Wu, 2004; Tang et al., 2012; Yang et al., 2014; Wang, 2016), while one study reported no adverse events in the treatment group but documented minor adverse events in the control group (Zhao, 2016). The incidence of adverse events was 4.49% (21/467) in the treatment group and 7.38% (34/461) in the control group.

As summarized in Table 4, the control group was associated with several adverse effects, including palpitations, abnormal body mass, dry cough, abdominal distension or constipation, abnormal liver function, nausea, fatigue, muscle pain, dizziness, mild drowsiness, sweating, rash, and gastrointestinal issues such as diarrhea or abdominal pain. In contrast, the treatment group exhibited adverse events such as palpitations, abnormal body mass, abdominal distension or constipation, abnormal liver function, nausea, fatigue, dizziness, rash, and gastrointestinal symptoms like diarrhea or abdominal pain. Notably, some of the adverse events reported in the studies were self-limiting. Specifically, in one study (Zhao, 2016), three mild cases of drowsiness and sweating in the control group spontaneously resolved. Additionally, another study (Huang, 2013) observed one case of upper abdominal discomfort and one case of nausea accompanied by facial flushing in the treatment group, which were mitigated by adjusting the timing of medication intake to after meals.

**TABLE 4:** Incidence rate of adverse events.

Type	Number of adverse events		Refs
	Experimental group	Control group	
Tachycardia/bradycardia/Palpitation	1	3	Xiong et al. (2013), Hong et al. (2017), Jiang et al. (2024)
Abnormal body mass	2	4	Hong et al. (2017)
Dry cough		2	Huang (2013)
Bloating/constipation	5	6	Wu et al., 2008; Huang (2013), Zhang (2021)
Abnormal liver Function	1	2	Wu et al. (2008)
Nausea	2	2	Wu et al. (2008), Huang (2013)
Lacking in strength	3	4	Wu et al. (2008), Xiong et al. (2013)
Myodynia		1	Wu et al. (2008)
Dizziness	3	4	Xiong et al. (2013), Jiang et al. (2024)
Tetter	2	1	Jiang et al. (2024)
Mild drowsiness and sweating		3	Zhao (2016)
Diarrhea/abdominal pain	2	2	Jiang et al. (2024)

## 4 Discussion

PLE represents one of the effective modalities for treating dyslipidemia. So far, numerous metabolites have been isolated and characterized from persimmon leaves, which are rich in phenolic metabolites, terpenoids, and other bioactive metabolites. Among these, flavonoids within the phenolic metabolites have been shown to play a pivotal role in improving the concentration of lipid metabolites. Studies have shown that increased dietary intake of flavonoids is associated with improved lipid profiles, particularly in women (Li et al., 2013). Furthermore, flavonoid intake has been inversely associated with the risk of CVD (Wang et al., 2014). NXQT, a film-coated tablet formulated from persimmon leaves, has undergone continuous refinement in both composition and manufacturing processes in recent years. Its primary metabolites include flavonoids, triterpenoids, and phenolic acid (Liu, 2023). Studies have demonstrated that quercetin and kaempferol, two prominent flavonoid metabolites, rank prominently in content and antioxidant activity within NXQT. This composition aligns with the primary therapeutic metabolites of Diospyros kaki leaves, thereby earning NXQT the distinction of being a CCPP derived from Diospyros kaki Leaves (Kazzem et al., 2019). Moreover, a variety of formulations containing PLE have been developed, including NXQC sustained-release tablets of persimmon leaf flavonoids (Liang et al., 2013), ointments formulated with persimmon leaf flavonoids (Zhao, 2015), orally disintegrating tablets (He et al., 2017), and so forth.

A multitude of cardiovascular and cerebrovascular diseases are frequently accompanied by dyslipidemia, which also serves as a potential high-risk factor for the onset and progression of numerous diseases (Silverman et al., 2016; Marston et al., 2019). The standardized management of blood lipids constitutes a pivotal strategy for both primary and secondary prevention of cardiovascular and cerebrovascular diseases (Cardiovascular Disease Branch of the Chinese Medical Association et al., 2020; Gai, 2020; Chinese Society of Cardiology et al., 2023). Atherosclerosis (AS) is a pivotal pathological foundation for cardiovascular and cerebrovascular diseases. The vascular disorders induced by AS encompass coronary heart disease, cerebrovascular disease, and peripheral artery disease, among others (Benjamin et al., 2017). Vascular endothelial dysfunction is a significant factor promoting the progression of atherosclerosis, resulting in decreased nitric oxide (NO) bioavailability and exacerbating atherosclerosis. Furthermore, hyperlipidemia can impair NO production by influencing oxidative stress, further disrupting vascular homeostasis (Förstermann et al., 2017). AS also promotes foam cell accumulation and plaque formation through inflammatory responses that damage macrophages (Maitra et al., 2009; Chinetti-Gbaguidi et al., 2011). It is noteworthy that animal experimental studies have indicated that PLE not only improves various lipid parameters but also inhibits the onset and progression of atherosclerosis through relevant mechanisms. Administration of PLE to rats maintained on a high-fat diet significantly decreased plasma concentrations of TC, TG, and LDL-C (Zhang et al., 2005; Pan et al., 2016; Wang et al., 2023a). At the molecular level, PLE directly targets key genes involved in lipid synthesis, effectively reducing excessive fat accumulation. In high-fat diet (HFD)-fed mice, PLE normalizes the overexpression of hepatic lipogenic genes, including FAS, SCD1, acetyl-CoA carboxylase (ACC), and sterol regulatory element-binding protein 1c (SREBP-1c). Research suggests that its regulatory effect may surpass that of simvastatin. Additionally, PLE downregulates other lipogenic genes and enzymes, including ATP-citrate lyase (ACL), phosphatidate phosphatase (PAP), diacylglycerol acyltransferase (DGAT), peroxisome proliferator-activated receptor γ (PPARγ), and 3-hydroxy-3-methylglutaryl-CoA (HMG-CoA) reductase (Jung et al., 2012; Kim et al., 2016). Through these actions, PLE inhibits hepatic fatty acid synthesis, thereby reducing lipid droplet formation in hepatocytes, as well as the secretion of triglycerides and cholesterol into the bloodstream. Beyond direct gene regulation, PLE can improve lipid metabolism by regulating the levels of short-chain fatty acids (SCFAs) in the intestines (Gao et al., 2025). Additionally, studies have shown that this extract can ameliorate lipid peroxidation in rats with hyperlipidemia, enhancing antioxidant capacity. It also effectively improves vascular endothelial cell function by modulating NO/ET and TXA2/PGI2 levels (Hu, 2012), and reduces intracellular cholesterol content by downregulating related proteins, thereby inhibiting lipid accumulation in macrophage-derived foam cells (Zhou, 2023). These findings further underscore the therapeutic potential of PLE. However, one study revealed that the oral administration of total flavonoids from persimmon leaves to apoE−/− mice fed a high-fat diet could inhibit the formation of atherosclerotic plaques in the thoracic and abdominal aorta, yet it failed to improve the body weight and blood lipid profiles of the mice. It was hypothesized that the anti-atherosclerotic effect of persimmon leaf flavonoids may be mediated by reducing serum malondialdehyde levels in mice (Chen et al., 2011b).

Although the efficacy and role of PLE have been extensively explored, systematic reviews and meta-analyses evaluating its use as an adjunctive therapy for dyslipidemia are lacking. Furthermore, there is limited evidence regarding its therapeutic application in other diseases. This study represents the first systematic evaluation of the efficacy of PLE in the adjunctive treatment of dyslipidemia. The analysis was conducted and reported in accordance with the PRISMA guidelines. Our study systematically synthesized 16 clinical studies on using PLE in managing dyslipidemia to assess the impact of PLE supplementation on adult lipid profiles. Through this rigorous scientific analysis, we have validated the potential of PLE to reduce serum TC, TG, and LDL-C, while increasing HDL-C levels, with statistically significant results maintained across all sensitivity analyses.

In our ongoing research, the incorporation of PLE demonstrated a more significant improvement in TC, TG, HDL-C, and LDL-C indices compared to guidelines that recommend routine treatment, exhibiting a positive synergistic effect. It is recognized, that certain biomedicine lipid-lowering therapies may exhibit higher efficacy in specific scenarios, particularly in reducing blood lipid indexes. In an RCT involving hyperlipidemia patients included in this study, the treatment group received NXQT combined with half-dose simvastatin (20 mg). By contrast, the control group received full-dose simvastatin (40 mg) (Wu et al., 2008). The results indicated no significant difference in the efficacy of reducing blood lipid indices between the two groups. However, the treatment group exhibited significantly fewer toxic side effects compared to the control group. This finding confirms the effectiveness of PLE in treating dyslipidemia and underscores its advantages of high safety and good tolerance. Furthermore, antipsychotic drugs used in the treatment of mental illness are associated with metabolic side effects, with metabolic syndrome occurring in approximately 40% of patients with chronic schizophrenia (Carli et al., 2021). Two studies in this analysis demonstrated that PLE, an adjunctive therapy for dyslipidemia induced by the antipsychotic olanzapine, effectively achieved lipid-lowering effects without compromising psychiatric symptom management (Hong et al., 2017; Liu, 2019).

Subgroup analyses were conducted to assess the potential impact of varying drug dosage forms and doses. In the subgroup analysis focusing on dosage forms, no statistically significant differences were observed in TC and TG levels among participants in the capsule group. This may be attributed to the diverse study designs and limited studies available. In the dose subgroup analysis, only studies examining the combined application of NXQT were analyzed due to the scarcity of studies. Notably, most subgroups demonstrated therapeutic effects that aligned with the overall findings.

Among the included studies, 11 reported a few adverse events, but no severe adverse reactions were observed. The results indicated that when PLE was used as an adjunctive therapy in combination with standard treatment, the number of reported adverse events was slightly lower compared to when only standard treatment was used, suggesting that PLE may offer potential benefits in terms of safety. However, the adverse events reported in the studies were complex and varied. In clinical studies of PLE for the treatment of other diseases, elevated levels of gamma-glutamyl transpeptidase, abnormal urine tests, and adverse events such as headaches have been reported during the treatment period (Liang et al., 2022; Du, 2022). It remains difficult to determine whether these effects were caused by PLE or by other factors. Therefore, more detailed studies on the adverse events associated with PLE are necessary to clarify its safety profile. Additionally, studies examining drug-drug interactions should also be considered to provide more comprehensive safety evidence for the clinical application of PLE.

Concurrently, we acknowledge that the present study has certain limitations. Firstly, the current meta-analysis encompasses a limited number of studies, each characterized by relatively small sample sizes. Secondly, substantial heterogeneity is evident among the included studies, potentially attributed to variations in trial design, patient demographics, disease severity, treatment dosages, and intervention durations. Third, the included RCTs exhibited certain methodological limitations, primarily manifested as inadequate reporting of key methodological elements, including randomization sequence generation, allocation concealment, and blinding implementation. These methodological shortcomings may potentially compromise the robustness of the study conclusions to some extent. Nevertheless, the available data still provide valuable reference evidence for evaluating the clinical efficacy and safety of PLE in the management of dyslipidemia. Fourthly, all the studies included in this research were conducted in China, potentially introducing cultural and regional bias.

In future studies, conducting a well-designed, rigorously controlled, and having sufficient statistical powered RCT is of paramount importance. The primary aim is to systematically and comprehensively evaluate the lipid-lowering effect, safety, and tolerability of PLE in patients with dyslipidemia. Such high-quality research will provide strong evidence for the potential application of PLE in treating hyperlipidemia. It should be emphasized that, in the design of future trials, special attention must be given to improving the methodological rigor of the study, particularly in the implementation of blind procedures and the management of allocation concealment. This will minimize the risk of bias, thereby enhancing the internal validity and scientific integrity of the study. Additionally, it is important to strengthen the in-depth exploration and analysis of safety-related data concerning PLE. While routine monitoring for common adverse events is important, special focus should also be placed on rare adverse events that may arise during long-term use, as well as on potential interactions between PLE and other lipid-lowering drugs or medications used to treat underlying conditions. Furthermore, for subgroups of patients with varying ages and comorbidities, any differences in the safety profile of PLE should be analyzed through stratification. These improvements will not only provide more comprehensive guidance for clinicians, ensuring patient safety, but also help clarify the clinical positioning of PLE and provide critical support for developing personalized treatment plans.

## 5 Conclusion

The findings of this study demonstrate that the adjunctive use of PLE in treating dyslipidemia, based on guidelines that recommend routine treatment, can effectively reduce TG, TC, and LDL-C levels while improving HDL-C. Given the notable heterogeneity among existing studies and the prevalent issue of low methodological quality, there is a pressing need for clinical trials that incorporate high methodological rigor, large sample sizes, and extended assessment of the efficacy of PLE in treating dyslipidemia.

## Data Availability

The original contributions presented in the study are included in the article/Supplementary Material, further inquiries can be directed to the corresponding authors.
