# Supplementary material for: Persimmon leaf extract in dyslipidemia: a systematic review and meta-analysis
Source: Front Pharmacol. 2025 Sep 15;16:1572678. doi: 10.3389/fphar.2025.1572678 (PMC12477231; doi:10.3389/fphar.2025.1572678)
Supplement: Supplementary file 1 [file Table1.docx]

**Supplementary table 1. search strategy**

| **#** | **Searches** |
| --- | --- |
| **Search strategies of CNKI (Searched from inception to Nov. 01, 2024 and found 230 literature)** | |
| **#1** | TKA %= ('脑心清'+'柿子叶提取物'+'柿叶黄酮'+'柿属植物') AND TKA %= ('随机对照试验'+'临床对照试验'+'随机'+'安慰剂'+'对照'+'盲法'+'试验'+'分组')  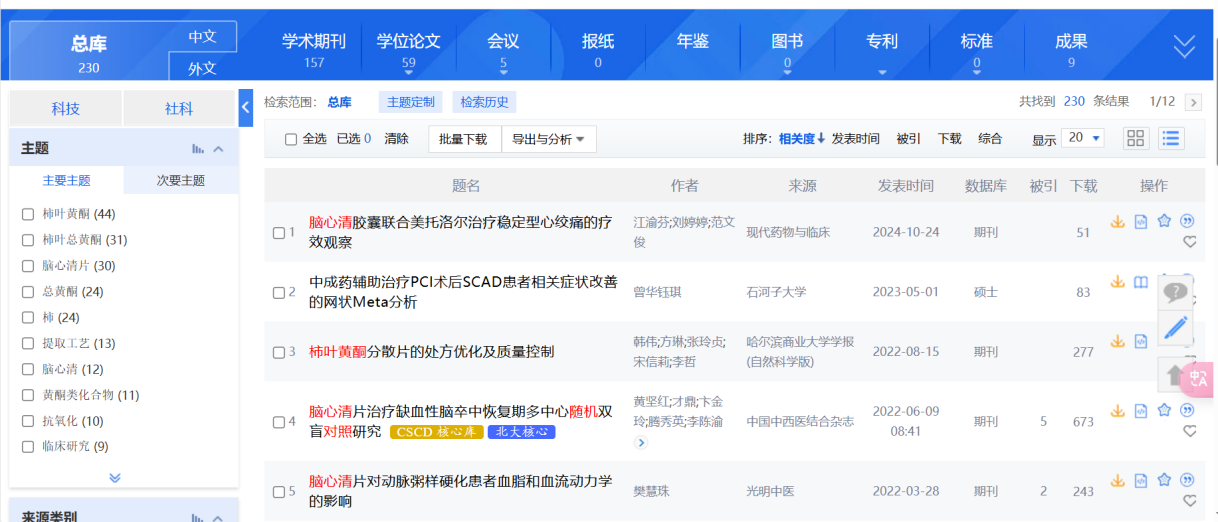 |
| **Search strategies of Wanfang (Searched from inception to Nov. 01, 2024 and found 152 literature)** | |
| **#1** | 主题: (“脑心清” or “柿子叶提取物” or “柿叶黄酮” or “柿属植物”) and (“随机对照试验” or “临床对照试验” or “随机” or “安慰剂” or “对照” or “盲法” or “试验” or “分组”)  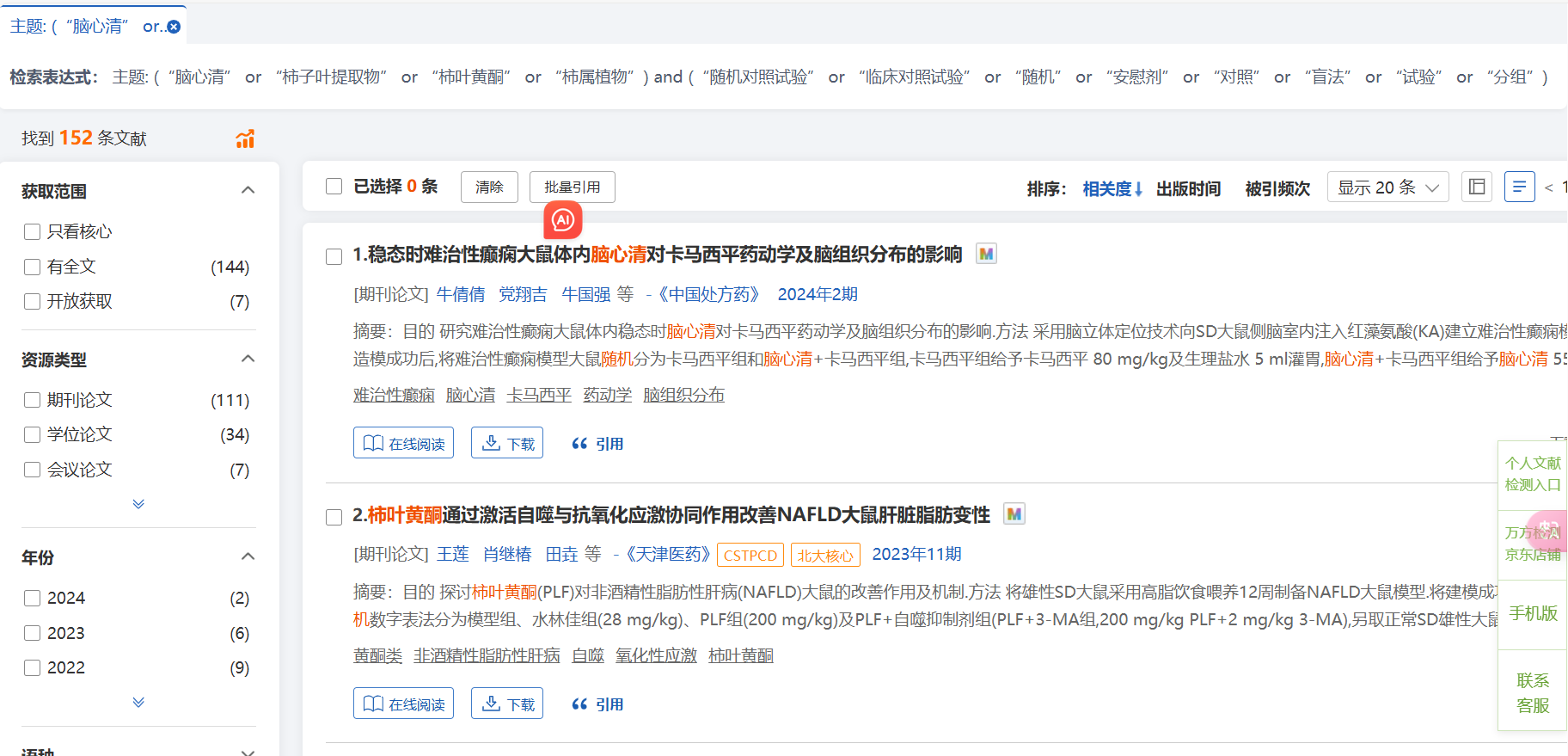 |
| **Search strategies of VIP (Searched from inception to Nov. 01, 2024 and found 123 literature)** | |
| **#1** | M=(脑心清 OR 柿子叶提取物 OR 柿叶黄酮 OR 柿属植物) AND R=(随机对照试验 OR 临床对照试验 OR 随机 OR 安慰剂 OR 盲法 OR 对照 OR 试验 OR 分组)  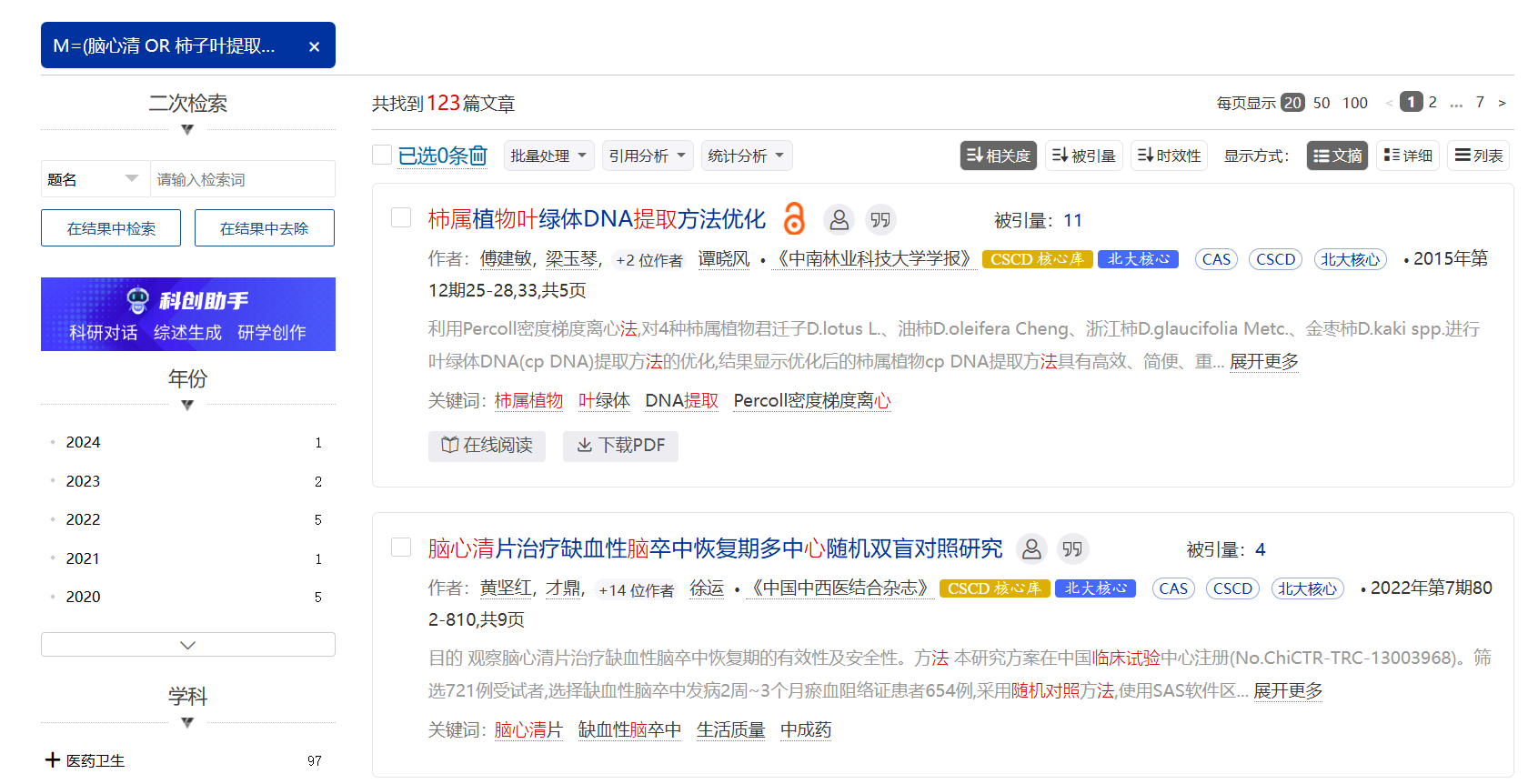 |
| **Search strategies of SinoMed (Searched from inception to Nov. 01, 2024 and found 80 literature)** | |
| **#1** | ("脑心清" OR "柿子叶提取物" OR "柿叶黄酮" OR "柿属植物") AND ("随机对照试验" OR "临床对照试验" OR "随机" OR "安慰剂" OR "盲法" OR "对照" OR "试验" OR "分组")  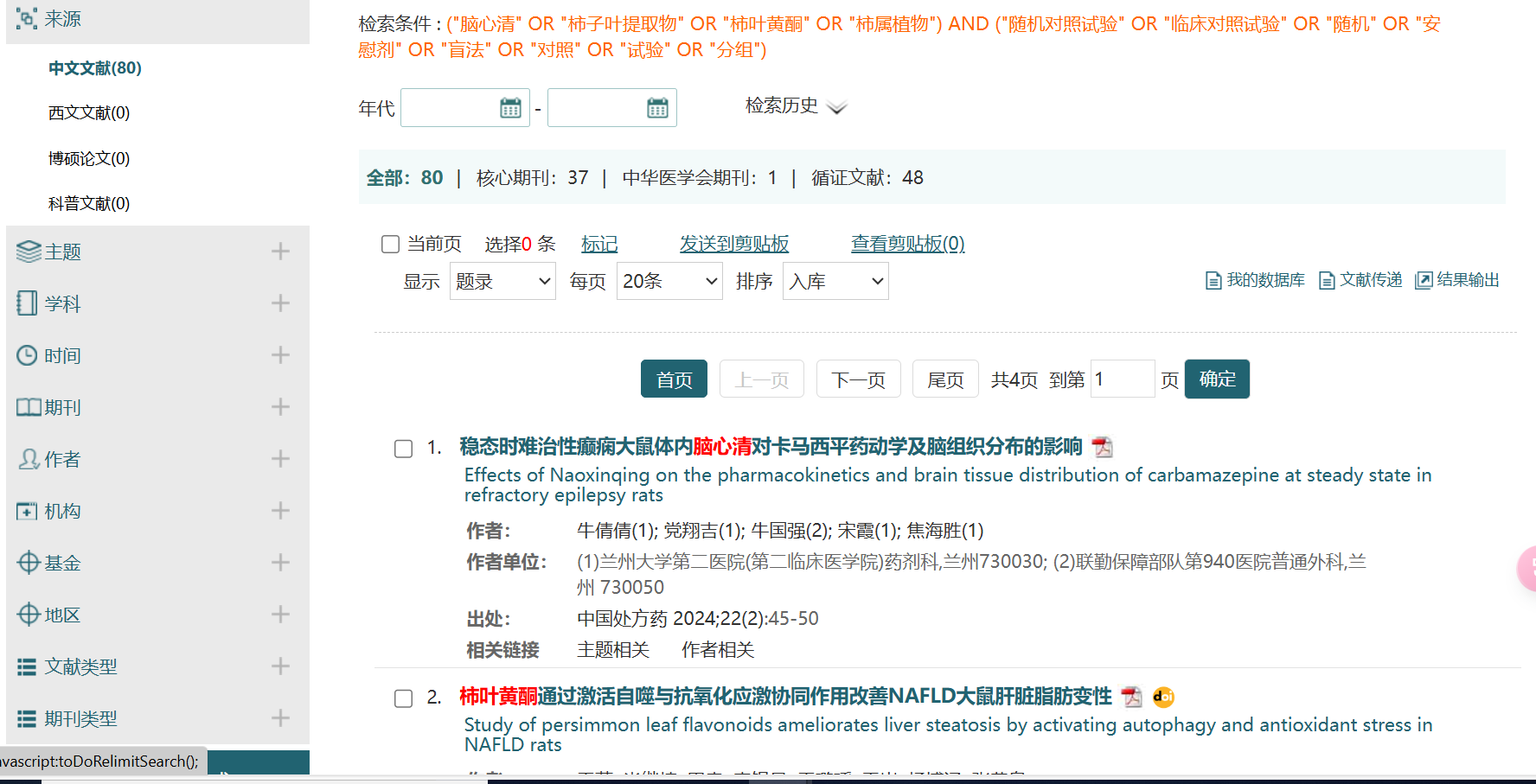 |
| **Search strategies of PubMed (Searched from inception to Nov. 01, 2024 and found 11 literature)** | |
| **#1** | (((Persimmon leaf extract[Title/Abstract]) OR (naoxinqing[Title/Abstract])) OR (persimmon flavones[Title/Abstract])) OR (diospyros[Title/Abstract])——820 |
| **#2** | (((((((controlled clinical trial[Title/Abstract]) OR (Clinical Trial[Title/Abstract])) OR (placebo[Title/Abstract])) OR (random[Title/Abstract])) OR (allocated[Title/Abstract])) OR (assign[Title/Abstract])) OR (RCT[Title/Abstract])) OR ("Randomized Controlled Trial" [Title/Abstract]) |
| **#3** | #1 AND #2——11  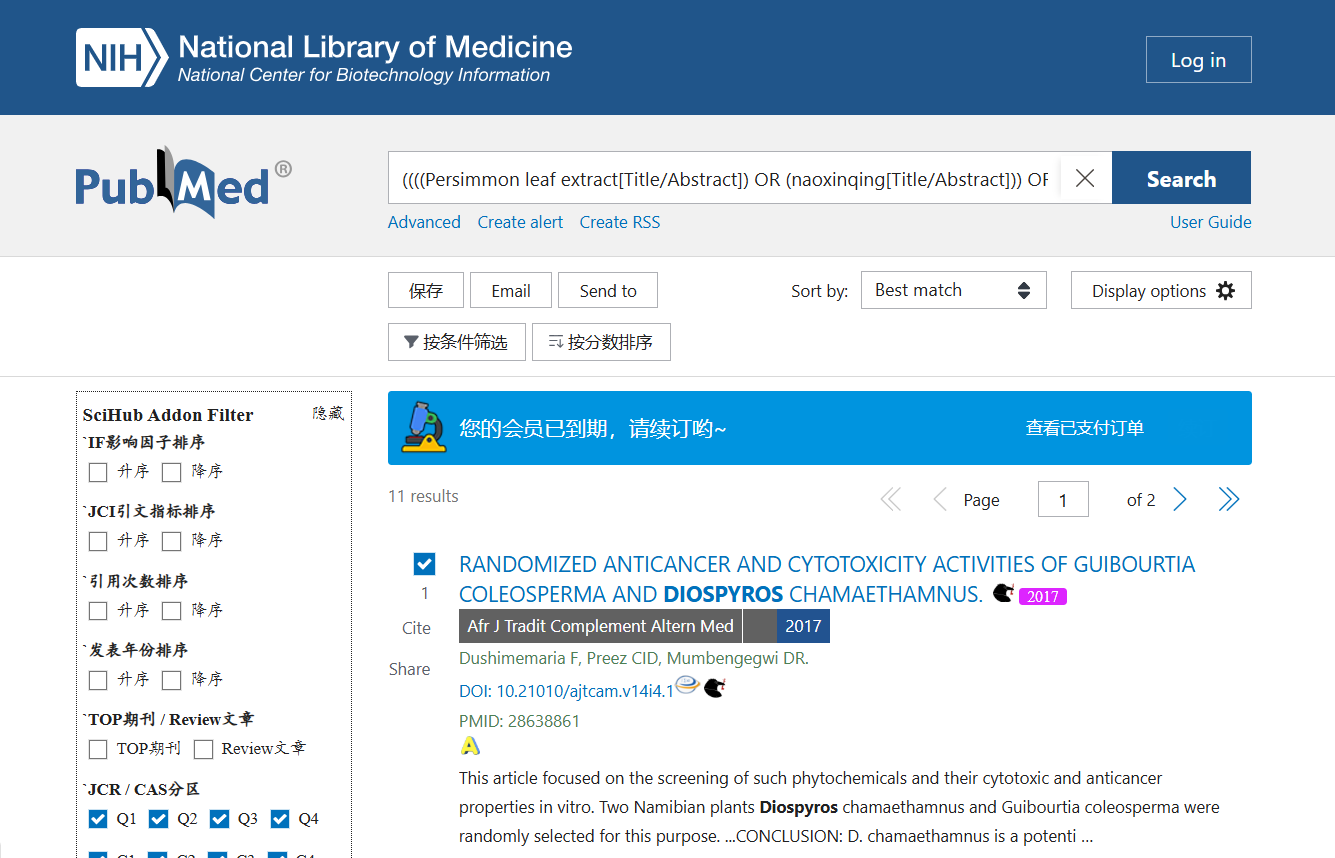 |
| **Search strategies of EMbase (Searched from inception to Nov. 01, 2024 and found 15 literature)** | |
| **#1** | 'Persimmon leaf extract':ti,ab OR 'naoxinqing':ti,ab OR 'persimmon flavones':ti,ab OR 'diospyros':ti,ab——969  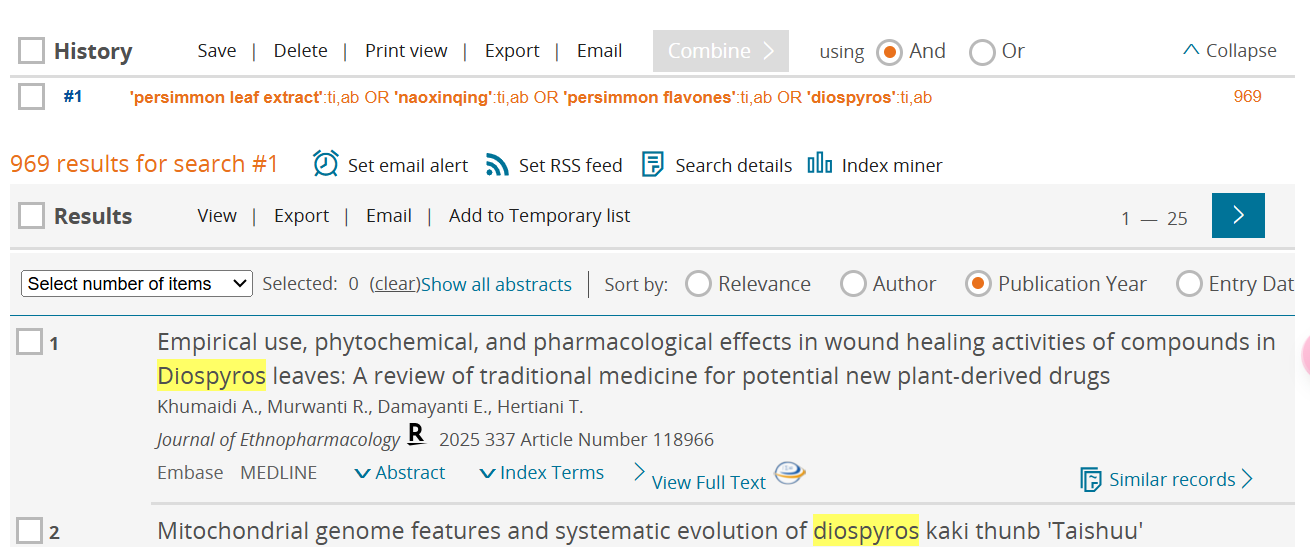 |
| **#2** | 'randomized controlled trial':ti,ab OR 'controlled clinical trial':ti,ab OR 'randomized':ti,ab OR 'placebo':ti,ab OR 'random*':ti,ab OR 'allocate*':ti,ab OR 'assign*':ti,ab OR 'RCT*':ti,ab |
| **#3** | #1 AND #2——15  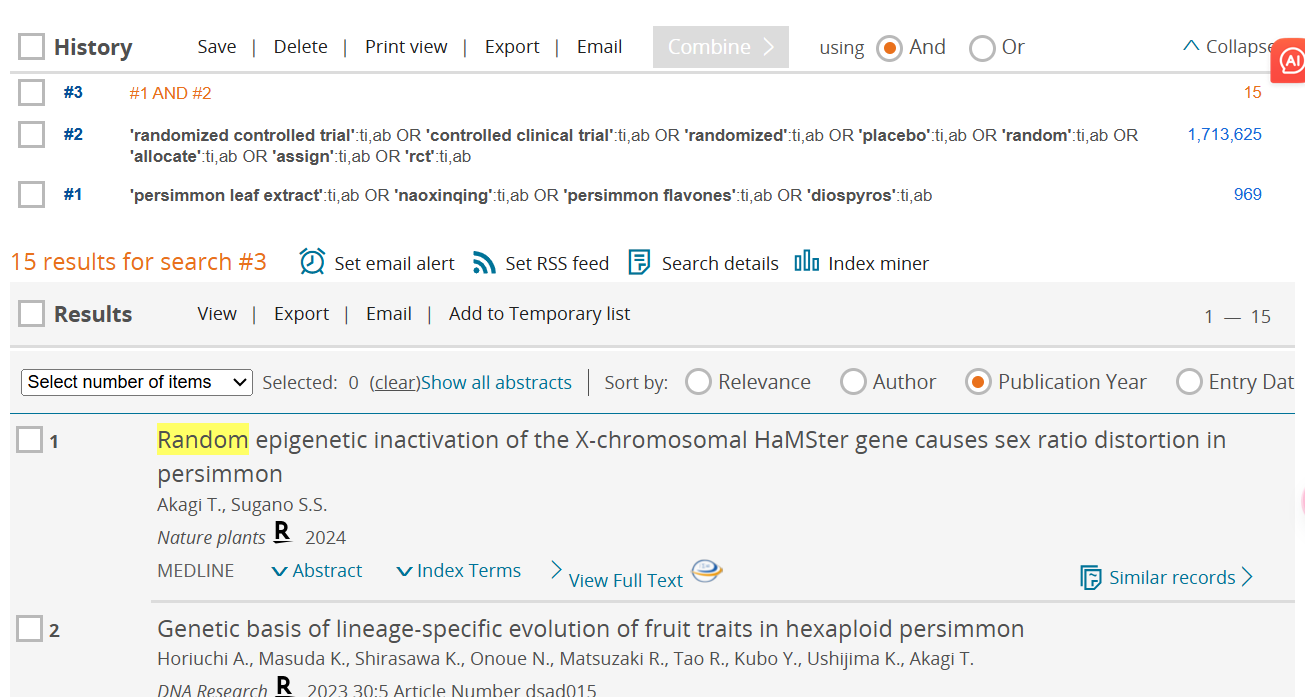 |
| **Search strategies of Web of Science (Searched from inception to Nov. 01, 2024 and found 76 literature)** | |
| **#1** | TS=(Persimmon leaf extract) OR (naoxinqing) OR (persimmon flavones) OR (diospyros) |
| **#2** | TS=(randomized controlled trial OR (controlled clinical trial) OR (randomized) OR (placebo) OR (random) OR (allocate) OR (MI) OR (assign) OR (RCT)) |
| **#3** | #1 AND #2  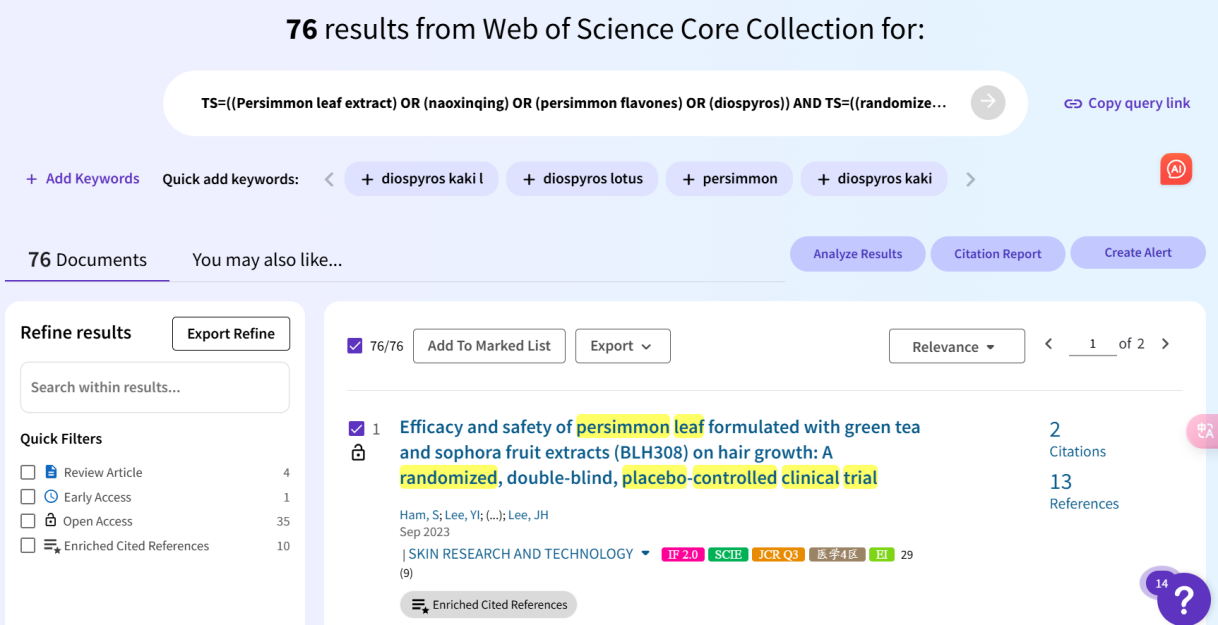 |
| **Search strategies of Cochrane Central Register of Controlled Trials (CENTRAL) (Searched from inception to Nov. 01, 2024 and found 12 literature)** | |
| **#1** | (Persimmon leaf extract):ti,ab,kw OR (naoxinqing):ti,ab,kw OR (persimmon flavones):ti,ab,kw OR (diospyros):ti,ab,kw |
| **#2** | (randomized controlled trial):ti,ab,kw OR (controlled clinical trial):ti,ab,kw OR (randomized):ti,ab,kw OR (placebo):ti,ab,kw OR (random):ti,ab,kw OR (allocate):ti,ab,kw OR (MI):ti,ab,kw OR (assign):ti,ab,kw OR (RCT):ti,ab,kw |
| **#3** | #1 AND #2  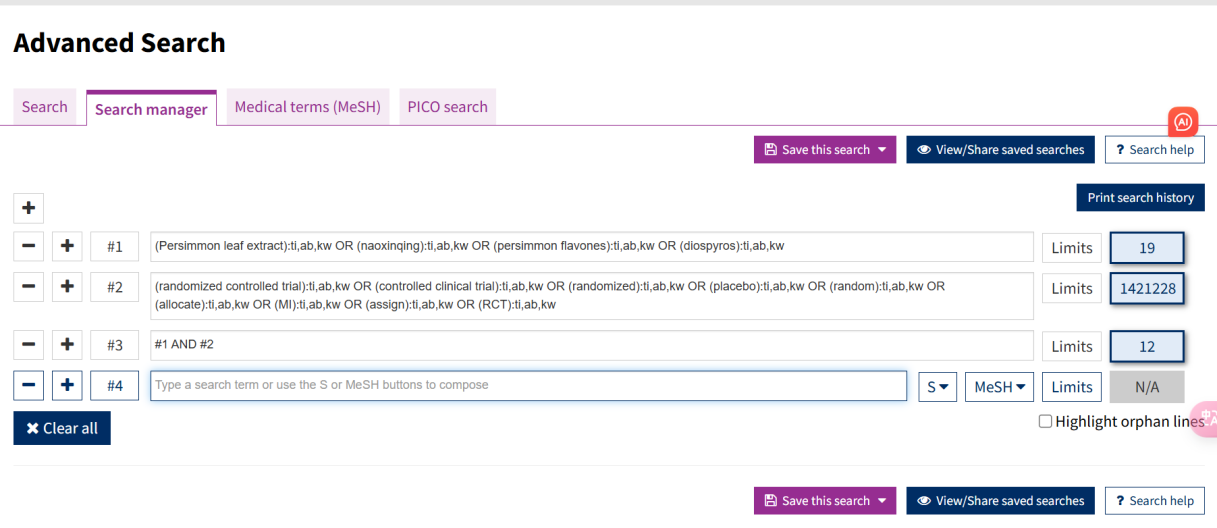 |
