# Supplementary material for: Persimmon leaf extract in dyslipidemia: a systematic review and meta-analysis
Source: Front Pharmacol. 2025 Sep 15;16:1572678. doi: 10.3389/fphar.2025.1572678 (PMC12477231; doi:10.3389/fphar.2025.1572678)
Supplement: Supplementary file 2 [file Table2.docx]

**Supplementary table 2. Commercial Preparation Summary Table**

| Study | Year | Clinical Trial  Design | Product Name | Dosage Form | Marketing Authorization | Manufacturer (Lot No.) | Batch Numbers |
| --- | --- | --- | --- | --- | --- | --- | --- |
| Hong | 2017 | RCT | Naoxinqing Tablet | Not Reported | NMPA Approval No. Z44021765, China | Guangzhou Baiyunshan Hutchison Whampoa Chinese Medicine Co., Ltd. | Not Reported |
| Huang | 2013 | RCT | Naoxinqing Tablet | Not Reported | Not Reported | Guangzhou Baiyunshan Hutchison Whampoa Chinese Medicine Co., Ltd. | Not Reported |
| Jiang | 2024 | RCT | Naoxinqing Capsule | Not Reported | Not Reported | Shenyang Dongxin Pharmaceutical Co., Ltd. | 20210520, 20220318, 20230209 |
| Liu | 2019 | RCT | Naoxinqing Tablet | Not Reported | Not Reported | Guangzhou Baiyunshan Hutchison Whampoa Chinese Medicine Co., Ltd. | Not Reported |
| Lv | 2020 | RCT | Naoxinqing Tablet | Not Reported | NMPA Approval No. Z44021765, China | Guangzhou Baiyunshan Hutchison Whampoa Chinese Medicine Co., Ltd. | Not Reported |
| Qiao | 2013 | RCT | Naoxinqing Tablet | Not Reported | Not Reported | Guangzhou Baiyunshan Hutchison Whampoa Chinese Medicine Co., Ltd. | Not Reported |
| Tang | 2012 | RCT | Naoxinqing Tablet | Not Reported | NMPA Approval No. Z44021765, China | Guangzhou Baiyunshan Hutchison Whampoa Chinese Medicine Co., Ltd. | C6A002 |
| Wang | 2016 | RCT | Naoxinqing Tablet | Not Reported | NMPA Approval No. Z44021765, China | Guangzhou Baiyunshan Hutchison Whampoa Chinese Medicine Co., Ltd. | Not Reported |
| Wei | 2018 | RCT | Naoxinqing Tablet | Not Reported | NMPA Approval No. Z44021765, China | Guangzhou Baiyunshan Hutchison Whampoa Chinese Medicine Co., Ltd. | Not Reported |
| Wu | 2004 | RCT | Naoxinqing Tablet | Not Reported | NMPA Approval No. Z44021765, China | Guangzhou Baiyunshan Hutchison Whampoa Chinese Medicine Co., Ltd. | Not Reported |
| Wu | 2008 | RCT | Naoxinqing Tablet | Not Reported | Not Reported | Guangzhou Baiyunshan Hutchison Whampoa Chinese Medicine Co., Ltd. | D7A005 |
| Xiong | 2013 | RCT | Naoxinqing Tablet | Not Reported | Not Reported | Not Reported | Not Reported |
| Yang | 2014 | RCT | Naoxinqing Tablet | Not Reported | Not Reported | Guangzhou Baiyunshan Hutchison Whampoa Chinese Medicine Co., Ltd. | Not Reported |
| Zhang | 2021 | RCT | Naoxinqing Capsule | Not Reported | NMPA Approval No. Z20050689, China | Shenyang Dongxin Pharmaceutical Co., Ltd. | Not Reported |
| Zhao | 2016 | RCT | Naoxinqing Tablet | Not Reported | Not Reported | Guangzhou Baiyunshan Hutchison Whampoa Chinese Medicine Co., Ltd. | Not Reported |
| Zhou | 2015 | RCT | Naoxinqing Tablet | Not Reported | NMPA Approval No. Z44021765, China | Guangzhou Baiyunshan Hutchison Whampoa Chinese Medicine Co., Ltd. | Not Reported |
