# Supplementary material for: Persimmon leaf extract in dyslipidemia: a systematic review and meta-analysis
Source: Front Pharmacol. 2025 Sep 15;16:1572678. doi: 10.3389/fphar.2025.1572678 (PMC12477231; doi:10.3389/fphar.2025.1572678)
Supplement: Supplementary file 3 [file Table3.docx]

**Supplementary table 3. Inclusion and exclusion criteria for the study**

| Study | Year | Clinical Trial Design | Population | Inclusion criteria | Exclusion criteria |
| --- | --- | --- | --- | --- | --- |
| Hong | 2017 | RCT | Dyslipidemia due to olanzapine | 1. Meets the diagnostic criteria for schizophrenia and the syndrome differentiation criteria of blood stasis syndrome as stipulated in the "Clinical Research Guidelines for Traditional Chinese Medicine New Drugs";  2. The disease duration is ≥ 6 months;  3. Age range is 20 to 60 years old;  4. Clear consciousness, stable vital signs, and certain expression ability. | 1. Patients with severe organic mental disorders or schizophrenia caused by other psychoactive substances;  2. Pregnant or lactating women;  3. Those with a recent history of major surgery or severe infection;  4. Those with other positive mental symptoms or mental disorders;  5. Those with primary abnormal glucose and lipid metabolism and those who have been taking medications that may affect the results of this study. |
| Huang | 2013 | RCT | Hypertension | Compliant with the hypertension diagnosis and classification standards of the World Health Organization/ Hypertension Alliance in 1999 | secondary hypertension |
| Jiang | 2024 | RCT | Stable angina pectoris | 1. The patient's symptoms and signs were evaluated according to the diagnostic criteria for stable angina pectoris in the "Guidelines for the Diagnosis and Treatment of Stable Coronary Heart Disease"; 2. Those who had no allergies or other serious adverse reactions to the drugs used in this study; 3. Those who were aware of and adhered to the research protocol. | 1. Patients with stage IV angina pectoris in cardiovascular disease (CCS);  2. Those requiring revascularization and other surgical treatments; 3. Those with hematological diseases, severe infectious diseases, or immunodeficiency;  4. Patients with liver or kidney dysfunction;  5. Malignant tumor patients;  6. Patients with other cardiovascular diseases. |
| Liu | 2019 | RCT | Dyslipidemia due to olanzapine | 1. Meets the diagnostic criteria for schizophrenia as defined in the ICD-10 Classification of Mental and Behavioral Disorders; 2. Age range: 16 to 65 years old; 3. Obtained written informed consent from the family guardian or legal representative. | 1. Those who already had metabolic syndrome before enrollment or had a family history of metabolic syndrome.  2. Users of psychoactive substances or non-addictive substances.  3. Suffering from neurological diseases or other serious physical illnesses. |
| Lv | 2020 | RCT | Acute cerebral infarction | 1. Meets the diagnostic criteria of the "Chinese Guidelines for the Diagnosis and Treatment of Acute Ischemic Cerebral Stroke 2010", and has been confirmed as acute cerebral infarction through MRI and CT examinations;  2. Has a clear state of consciousness and no allergy to the study drug. | 1. Those with impaired consciousness and allergic to the drugs in this study;  2. Those with diabetes, impaired liver and kidney function, and gastrointestinal diseases;  3. Those with severe blood system diseases. |
| Qiao | 2013 | RCT | Coronary heart disease | Patients with coronary heart disease who were treated at the researcher's hospital. | Not described |
| Tang | 2012 | RCT | diabetic cerebral infarction | 1. Fasting blood glucose ≥ 7 mmol/L or 2-hour post-meal blood glucose ≥ 11 mmol/L during the oral glucose tolerance test;  2. Age 18 - 75 years old, gender not restricted, blood pressure < 140/90 mmHg;  3. MRI or CT indicates cerebral infarction;  4. After being selected, blood glucose should be well controlled, with fasting blood glucose ≤ 7 mmol/L, 2-hour post-meal blood glucose ≤ 11 mmol/L, urine ketone bodies (-), and cerebral infarction in a stable state. | 1. Patients with hypertension;  2. Patients with cerebral hemorrhage and those in the acute stage of cerebral infarction;  3. Severe infections;  4. Severe liver and kidney disorders and heart dysfunction;  5. Patients with severe acute diabetic complications;  6. Pregnant and lactating women;  7. Those who cannot cooperate or refuse to cooperate for other reasons. |
| Wang | 2016 | RCT | lower extremity atherosclerotic occlusive disease | 1. Diagnosed according to the diagnostic criteria for arteriosclerotic occlusive disease specified by the Peripheral Vascular Disease Professional Committee of the Chinese Association of Traditional and Western Medicine in 2005 (revised version);  2. Age ≥ 60 years;  3. General condition is acceptable, and no other severe organic or systemic diseases such as heart failure are coexisting;  4. Informed consent. | 1. With severe liver and kidney dysfunction;  2. Indications for interventional treatment;  3. Severe brain and neurological functional disorders;  4. History of allergies;  5. Having experienced major illness episodes, severe trauma or major surgeries within the past 6 months;  6. Severe primary limb functional disorders. |
| Wei | 2018 | RCT | hyperlipidemia | Compliant with the diagnostic criteria for hyperlipidemia in the "2007 Chinese Guidelines for the Prevention and Treatment of Hyperlipidemia" | Nephrotic syndrome, hypothyroidism, acute and chronic liver and gallbladder diseases, drug-induced hyperlipidemia, homozygous familial hypercholesterolemia, abnormal liver function, severe cardiovascular and cerebrovascular diseases, severe trauma and surgery, pregnant or lactating women, alcoholics. |
| Wu | 2004 | RCT | Coronary heart disease | According to the diagnostic criteria for coronary heart disease of the WHO in 1979 and the revised "Diagnostic Criteria for Coronary Heart Disease" by the National Symposium on Coronary Heart Disease Angina Pectoris, the classification diagnosis is based on the opinions of the World Health Organization regarding "Nomenclature and Diagnostic Criteria for Ischemic Heart Disease" | Not described |
| Wu | 2008 | RCT | hyperlipidemia | According to the diagnostic criteria for lipid-regulating drugs formulated in the "Guidelines for Clinical Research of New Traditional Chinese Medicines" issued by the Ministry of Health. Patients who have discontinued the drugs used to treat high blood lipids, after undergoing dietary education for 2 to 4 weeks, have their blood drawn for fasting tests. Anyone whose serum total cholesterol (TC) is ≥ 5.85 mmol/L, triglycerides (TG) are ≥ 1.98 mmol/L, and high-density lipoprotein cholesterol (HDL-C) for men is ≤ 1.04 mmol/L and for women is ≤ 1.17 mmol/L, meeting any one or more of these criteria, can be regarded as an observation subject. | Nephrotic syndrome, hypothyroidism, acute and chronic liver and gallbladder diseases, drug-induced hyperlipidemia, homozygous familial hypercholesterolemia, abnormal liver function, severe cardiovascular and cerebrovascular diseases, severe trauma and surgery, pregnant or lactating women, alcoholics. |
| Xiong | 2013 | RCT | Unstable angina pectoris | The elderly patients with unstable angina pectoris who were admitted to the researcher's hospital. | Exclude patients with liver dysfunction, diabetes, infectious diseases, tumors, etc., and those with a history of drug allergies. |
| Yang | 2014 | RCT | hypertension | 1. According to the diagnostic criteria of the World Health Organization/International Society of Hypertension (WHO/ISH) in 1999, and based on the patient's medical history, clinical manifestations, blood pressure measurement, laboratory and imaging examinations, secondary hypertension was excluded;  2. Age 30 - 60 years old;  3. Blood pressure levels: grades 1 and 2, cardiovascular risk stratification as low-risk and moderate-risk;  4. No history of hypertension in the past, and this was the first diagnosis of hypertension upon admission;  5. Willing to participate in the treatment observation. | 1. Those who do not meet the above diagnostic criteria for hypertension;  2. Those with other diseases: such as severe diseases of the heart, brain, lungs, liver, kidneys, and diabetes patients;  3. Those who failed to adhere to the doctor's advice and take the prescribed medication regularly within 6 months;  4. Those with contraindications to valsartan and the drugs involved in this research project;  5. Those with chronic inflammatory diseases such as rheumatic diseases or asthma, acute inflammatory diseases, tumors, etc. |
| Zhang | 2021 | RCT | Cerebral arteriosclerosis | 1. Age over 45 years old;  2. Initial symptoms of advanced neurological instability and/or diffuse brain damage;  3. Manifestations of systemic arteriosclerosis, such as grade II or above of fundus arteriosclerosis or widened aortic arch and harder temporal artery or radial artery, as well as coronary heart disease, etc.;  4. Positive neurological signs, asymmetric tendon reflexes, positive palmar-mental reflex and/or sucking reflex;  5. Elevated serum cholesterol;  6. Exclude other brain diseases. | 1. Patients with other neurological disorders;  2. Patients with diabetes or liver/kidney diseases;  3. Patients with low blood pressure or those allergic to the test drug. |
| Zhao | 2016 | RCT | Chronic cerebral circulatory insufficiency | Adopt the CCCI diagnostic criteria determined at the 2000 Japanese Stroke Conference:  1. The patient has symptoms such as headache, severe dizziness, and dizziness;  2. Neurological examinations show that the patient has no neurological signs related to brain localization;  3. Relevant examinations indicate that the patient has arteriosclerosis, such as high blood pressure and changes in the retinal arteries;  4. Head MRI or CT examinations show that the patient has no vascular organic brain lesions;  5. The patient's above symptoms are not caused by other reasons;  6. The patient's age is 55 years or above, and cerebral blood flow is significantly reduced. Through cranial Doppler ultrasound and digital subtraction angiography techniques, it can be found that the cerebral perfusion arteries are occluded or have a narrowing phenomenon. | 1. Tumors, various acute and chronic inflammations, patients without any organic changes such as brain vascular disorders as detected by CT examination;  2. Patients with mental disorders, history of cerebral hemorrhage, or history of cerebral infarction. |
| Zhou | 2015 | RCT | Acute ischemic cerebral disease | The diagnostic criteria and scoring methods revised at the 4th National Academic Conference on Cerebrovascular Diseases in 1995, and confirmed by head CT and/or MRI, were implemented after all patients signed the informed consent form and were approved by the hospital's ethics committee. | Patients with cardiac function classified as NYHA Class III and those with severe hepatic or renal dysfunction. |
