# Supplementary material for: Persimmon leaf extract in dyslipidemia: a systematic review and meta-analysis
Source: Front Pharmacol. 2025 Sep 15;16:1572678. doi: 10.3389/fphar.2025.1572678 (PMC12477231; doi:10.3389/fphar.2025.1572678)
Supplement: Supplementary file 4 [file Table4.docx]

**Supplementary table 4. AMSTAR 2**

| **1. Did the research questions and inclusion criteria for the review include the components of**  **PICO?**   \| For Yes: Optional (recommended)  □ Timeframe for follow-up ☑ Yes  □ No  □ Population  □ Intervention  □ Comparator group  □ Outcome \| \| --- \|   **2. Did the report of the review contain an explicit statement that the review methods were**  **established prior to the conduct of the review and did the report justify any significant deviations from the protocol?** | |
| --- | --- | --- |
| 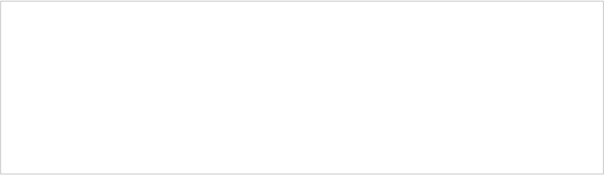For Yes:  For Partial Yes:  The authors state that they had a written protocol or guide that included ALL the following:  □ review question(s)  □ a search strategy  □ inclusion/exclusion criteria  □ a risk of bias assessment  □ Partial Yes □ No  As for partial yes, plus the protocol should be registered and should also have specified:  ☑ Yes  □ a meta-analysis/synthesis plan, if appropriate, *and*  □ a plan for investigating causes of heterogeneity  □ justification for any  deviations from the protocol | |
| **3. Did the review authors explain their selection of the study designs for inclusion in the review?** | |
| 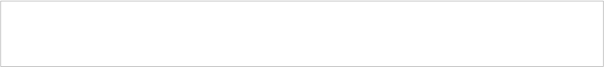For Yes, the review should satisfy ONE of the following:  □ *Explanationfor* including only RCTs  ☑ Yes □ No  □ OR *Explanationfor* including only NRSI  □ OR *Explanationfor* including both RCTs and NRSI | |
| **4. Did the review authors use a comprehensive literature search strategy?** | |
| 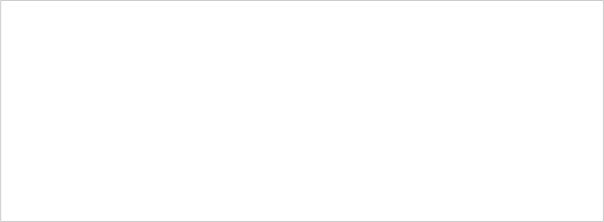For Yes, should also have (all the following):  For Partial Yes (all the following):  □ searched at least 2 databases  (relevant to research question)  □ provided keyword and/or search strategy  □ justified publication  restrictions (eg, language)  □ searched the reference  ☑ Yes  □ Partial Yes □ No  lists/bibliographies of included studies  □ searched trial/study registries  □ included/consulted content experts in the field  □ where relevant, searched for grey literature  □ conducted search within 24  months of completion of the review | |
| **5. Did the review authors perform study selection in duplicate?** | |
| 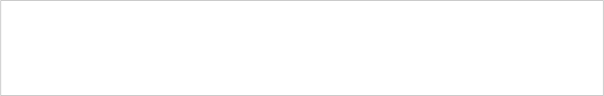For Yes, either ONE of the following:  ☑ Yes □ No  □ at least two reviewers independently agreed on selection of eligible studies and achieved consensus on which studies to include  □ OR two reviewers selected a sample of eligible studies and achieved good agreement (at least 80 per cent), with the remainder selected by one reviewer | |
| **6. Did the review authors perform data extraction in duplicate?** | |
| For Yes, either ONE of the following:  □ at least two reviewers achieved consensus on which data to extract  ☑ Yes |  |

| 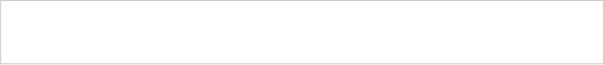from included studies □ No  □ OR two reviewers extracted data from a sample of eligible studies and achieved good agreement (at least 80 per cent), with the remainder  extracted by one reviewer |
| --- |
| **7. Did the review authors provide a list of excluded studies and justify the exclusions?** |
| 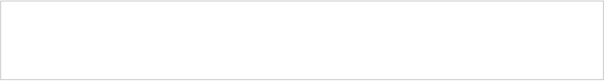For Partial Yes:  For Yes, must also have:  □ Justified the exclusion from the review of each  potentially relevant study  □ provided a list of all  ☑ Yes  □ Partial Yes □ No  potentially relevant studies  that were read in full text form but excluded from the review |
| **8. Did the review authors describe the included studies in adequate detail?** |
| 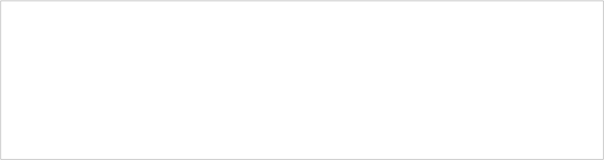For Yes, should also have ALL the following:  For Partial Yes (ALL the following):  □ described populations  □ described interventions  □ described comparators  □ described outcomes  □ described research designs  ☑ Yes  □ Partial Yes □ No  □ described population in detail  □ described intervention and comparator in detail  (including doses where relevant)  □ described study’s setting  □ timeframe for follow-up |
| **9. Did the review authors use a satisfactory technique for assessing the risk of bias (RoB) in**  **individual studies that were included in the review?**   \| **RCTs**  Yes  Partial Yes No  Includes only NRSI  ☑  □ □ □  For Partial Yes, must have assessed RoB from  For Yes, must also have assessed RoB from:  □ allocation sequence that was not truly random, *and*  □ selection of the reported  result from among multiple measurements or analyses of a specified outcome  □ unconcealed allocation, *and*  □ lack of blinding of patients  and assessors when assessing outcomes (unnecessary for  objective outcomes such as all cause mortality) \| \| --- \| \| **NRSI**  For Partial Yes, must have assessed RoB:  For Yes, must also have assessed RoB:  □ methods used to ascertain  exposures and outcomes,  *and*  □ selection of the reported  result from among multiple measurements or analyses of a specified outcome  □ Yes  □ Partial Yes □ No  □ Includes only RCTs  □ from confounding, *and*  □ from selection bias \|   **10. Did the review authors report on the sources of funding for the studies included in the review?** |
| 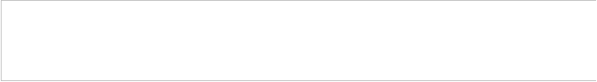For Yes  □ Must have reported on the sources of funding for individual studies included ☑ Yes  in the review. Note: Reporting that the reviewers looked for this information □ No  but it was not reported by study authors also qualifies |
| **11. If meta-analysis was performed did the review authors use appropriate methods for statistical**  **combination of results?** |
| \| **RCTs**  ☑ Yes □ No  □ No meta-analysis  For Yes:  □ The authors justified combining the data in a meta-analysis  □ AND they used an appropriate weighted technique to combine study results and adjusted for heterogeneity if present \| \| --- \| |

| □ AND investigated the causes of any heterogeneity conducted | | |  |
| --- | --- | --- | --- |
| **For NRSI**  For Yes:  □ The authors justified combining the data in a meta-analysis □ Yes  □ AND they used an appropriate weighted technique to combine □ No  study results, adjusting for heterogeneity if present □ No meta-analysis  □ AND they statistically combined effect estimates from NRSI conducted  that were adjusted for confounding, rather than combining  raw data, or justified combining raw data when adjusted effect estimates were not available  □ AND they reported separate summary estimates for RCTs and NRSI separately when both were included in the review | | |  |
| **12. If meta-analysis was performed, did the review authors assess the potential impact of RoB in**  **individual studies on the results of the meta-analysis or other evidence synthesis?** | | | |
|  | For Yes:  □ included only low risk of bias RCTs  □ OR, if the pooled estimate was based on RCTs and/or NRSI at variable RoB, the authors performed analyses to investigate possible impact of RoB on summary estimates of effect | ☑ Yes □ No  □ No meta-analysis conducted |  |
| **13. Did the review authors account for RoB in individual studies when interpreting/discussing**  **the results of the review?** | | | |
| 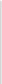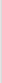For Yes:  ☑ Yes □ No  □ included only low risk of bias RCTs  □ OR, if RCTs with moderate or high RoB, or NRSI were included the  review provided a discussion of the likely impact of RoB on the results  **14. Did the review authors provide a satisfactory explanation for, and discussion of, any**  **heterogeneity observed in the results of the review?** | | | |
| 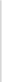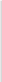For Yes:  ☑ Yes □ No  □ There was no significant heterogeneity in the results  □ OR if heterogeneity was present the authors performed an investigation of sources of any heterogeneity in the results and discussed the impact of this on the results of the review  **15. If they performed quantitative synthesis did the review authors carry out an adequate**  **investigation of publication bias (small study bias) and discuss its likely impact on the results of the review?** | | | |
|  | For Yes:  □ performed graphical or statistical tests for publication bias and  discussed the likelihood and magnitude of impact of publication bias | ☑ Yes □ No  □ No meta-analysis conducted |  |
| **16. Did the review authors report any potential sources of conflict of interest, including any**  **funding they received for conducting the review?** | | | |
|  | For Yes:  □ The authors reported no competing interests OR  □ The authors described their funding sources and how they managed potential conflicts of interest | ☑ Yes □ No |  |
